# HMGB1-mediated elevation of KLF7 facilitates hepatocellular carcinoma progression and metastasis through upregulating TLR4 and PTK2

**DOI:** 10.7150/thno.84388

**Published:** 2023-07-14

**Authors:** Weibo Feng, Jie Chen, Wenjie Huang, Guodong Wang, Xilang Chen, Lili Duan, Yue Yin, Xiaoping Chen, Bixiang Zhang, Mengyu Sun, Xiangyuan Luo, Yongzhan Nie, Daiming Fan, Kaichun Wu, Limin Xia

**Affiliations:** 1 State Key Laboratory of Holistic Integrative Management of Gastrointestinal Cancers and National Clinical Research Center for Digestive Diseases, Xijing Hospital of Digestive Diseases, Fourth Military Medical University, Xi'an 710032, Shaanxi Province, China.; 2 Department of Gastroenterology, Institute of Liver and Gastrointestinal Diseases, Hubei Key Laboratory of Hepato-Pancreato-Biliary Diseases, Tongji Hospital of Tongji Medical College, Huazhong University of Science and Technology, Wuhan 430030, Hubei Province, China.; 3 Hubei Key Laboratory of Hepato-Pancreato-Biliary Diseases; Hepatic Surgery Center, Tongji Hospital, Tongji Medical College, Huazhong University of Science and Technology; Clinical Medicine Research Center for Hepatic Surgery of Hubei Province; Key Laboratory of Organ Transplantation, Ministry of Education and Ministry of Public Health, Wuhan, Hubei, 430030, China.

**Keywords:** krüppel-like factor 7, toll-like receptor 4, protein tyrosine kinase 2, high mobility group box 1, hepatocellular carcinoma

## Abstract

**Background:** Metastasis is a major cause of HCC-related deaths with no effective pharmacotherapies. Chronic inflammation promotes HCC dissemination, however, its underlying mechanisms are not fully understood. Here, we investigated the role of Krüppel-like factor 7 (KLF7) in inflammation-provoked HCC metastasis and proposed therapeutic strategies for KLF7-positive patients.

**Methods:** The expression of KLF7 in human HCC specimens were examined by immunohistochemistry and quantitative real-time PCR. The luciferase reporter assays and chromatin immunoprecipitation assays were conducted to explore the transcriptional regulation related to KLF7. Orthotopic xenograft models and DEN/CCl_4_-induced HCC models were established to evaluate HCC progression and metastasis.

**Results:** KLF7 overexpression promotes HCC metastasis through transactivating toll-like receptor 4 (TLR4) and protein tyrosine kinase 2 (PTK2) expression. High mobility group box 1 (HMGB1) upregulates KLF7 expression through the TLR4/advanced glycosylation end-product specific receptor (RAGE)-PI3K-AKT-NF-κB pathway, forming an HMGB1-KLF7-TLR4 positive feedback loop. The HMGB1-KLF7-TLR4/PTK2 axis is gradually activated during the progression of inflammation-HCC transition. Genetic depletion of KLF7 impedes HMGB1-mediated HCC progression and metastasis. The combined application of TLR4 inhibitor TAK-242 and PTK2 inhibitor defactinib alleviates HCC progression and metastasis induced by the HMGB1-KLF7 axis. In human HCCs, KLF7 expression is positively correlated with cytoplasmic HMGB1, p-p65, TLR4, and PTK2 levels, and patients positively co-expressing HMGB1/KLF7, p-p65/KLF7, KLF7/TLR4 or KLF7/PTK2 exhibit the worst prognosis.

**Conclusions:** HMGB1-induced KLF7 overexpression facilitates HCC progression and metastasis by upregulating TLR4 and PTK2. Genetic ablation of KLF7 via AAV gene therapy and combined blockade of TLR4 and PTK2 represents promising therapy strategies for KLF7-positive HCC patients.

## Introduction

Hepatocellular carcinoma (HCC) is a lethal malignancy with a dismal prognosis, which remains a global health challenge lacking effective intervention 1. Although the evolution of systemic therapies has enriched therapeutic drugs for unresectable HCC, patients commonly receive limited benefits owing to the low objective response rate (ORR) and acquired resistance 2. High genetic heterogeneity of HCC is considered the primary cause of treatment failure, and combination therapies have been proposed to overcome this obstacle 3. Recently, the combination of atezolizumab and bevacizumab has become the first-line treatment for HCC instead of sorafenib. Several ongoing clinical trials of combination therapy also show encouraging results in improving HCC prognosis, indicating the exciting prospect of combination strategies in future HCC treatment 1. Currently, early recurrence and distant metastasis remain major hurdles in the clinical management of HCC. Hence, it is necessary to further elucidate the molecular mechanisms underlying HCC metastasis and propose feasible combination strategies for certain HCC subpopulations based on specific molecular signatures.

Dysregulated transcription factors that shape the aberrant cancer transcriptome are significant drivers of tumor progression and metastasis, thus representing a unique class of therapeutic targets 4. Krüppel-like factors (KLFs), a family of zinc finger-containing transcription factors with 17 members in humans, are involved in the onset and progression of human cancers by regulating various cancerous phenotypes 5. We profiled the expression patterns of KLF genes in paired HCC tissues and identified KLF7 as the most upregulated gene that significantly promoted HCC cell migration and proliferation. KLF7 is a well-defined oncogenic factor and an unfavorable prognostic biomarker in pancreatic ductal adenocarcinoma (PDAC), endometrial cancer and glioma 6-8. A recent study reported that KLF7 is up-regulated in human HCC and enhances tumor proliferation and invasion 9. Nevertheless, the functional role of KLF7 in HCC metastasis remains elusive, and further exploration of its oncogenic mechanisms in HCC may lead to new translational discoveries in HCC diagnosis and treatment.

The feedforward cycle of inflammation and hepatocyte death plays a critical role in the pathogenesis of chronic liver disease and HCC 10. HMGB1, a non-histone chromatin-associated protein, can be passively released from necrotic cells or actively secreted from immunocompetent cells during liver injury and inflammation 11. As a typical damage-associated molecular pattern (DAMP), extracellular HMGB1 transmits danger signals to neighboring cells by interacting with its classical receptors, including Toll-like receptors 2/4/9 (TLR2/4/9) and receptor for advanced glycation end products (RAGE) 12. Numerous studies have suggested that inflammation-provoked HMGB1 release promotes HCC initiation, progression and metastasis via multiple mechanisms 13-15. Here, we found that extracellular HMGB1 induced KLF7 upregulation in HCC cells and explored the functional role of KLF7 in HMGB1-mediated HCC development and metastasis.

In this study, we reported that HMGB1-induced KLF7 overexpression facilitated HCC development and metastasis through upregulating TLR4 and protein tyrosine kinase 2 (PTK2). Adeno-associated virus 8 (AAV8)-mediated *KLF7* knockout in hepatocytes or combination of TLR4 inhibitor TAK-242 and PTK2 antagonist defactinb effectively suppressed HMGB1-KLF7 axis-driven HCC development and metastasis.

## Materials and Methods

### Establishment of Orthotopic HCC Models

Male BALB/C nude mice (5 weeks old) were fed under standard conditions and cared for in accordance with the institutional guidelines for animal care. For orthotopic implantation, 50 µL PBS/Matrigel mixture with 2×10^6^ HCC cells was injected into the left liver lobe of mice via an 8 mm epigastric incision under anesthesia. *In vivo* liver tumor development and metastasis were monitored using bioluminescence. To detect *in vivo* signals, mice were intraperitoneally injected with D-luciferin (Xenogen, Hopkinton, MA, USA) at a dose of 100 mg/kg, and bioluminescence was recorded using an IVIS 100 Imaging System (Xenogen). The survival of the mice was recorded daily. After 9 weeks, the mice were sacrificed and their lungs were dissected for standard histological examination. These animal experiments were approved by the Committee on the Use of Live Animals in Teaching and Research (CULATR), Fourth Military Medical University.

### DEN/CCl_4_-induced HCC models

For chemically induced HCC, male C57BL/6 mice were intraperitoneally injected with DEN (25mg/kg) at week 2 postpartum and subsequently injected weekly with CCl_4_ (0.5 mL/kg, i.p., diluted with corn oil) from 4-week-old to 15-week-old. Mice were sacrificed at 34 weeks for statistical analysis or raised until death for survival analysis. All animal experiments were approved by the Committee on the Use of Live Animals in Teaching and Research (CULATR), Fourth Military Medical University.

Additional materials and methods are available in the online supplementary materials.

## Results

### KLF7 upregulation predicts poor clinical outcomes and high metastatic potentials in human HCC

Dysregulated *KLF* genes contribute to cancer development and metastasis 5. To identify dysregulated KLFs in HCC, we analyzed the expression profiles of *KLF* genes in HCC samples. Compared to para-tumor specimens, the mRNA levels of *KLF5*, *KLF7*, *KLF8*, and *KLF13* were elevated, whereas those of *KLF4*, *KLF6,* and *KLF10* were reduced in HCC specimens. The expressions of *KLF2*, *KLF3*, *KLF9*, *KLF11*, *KLF12*, *KLF15*, and *KLF16* did not differ between adjacent non-tumor tissues and HCC tissues. Moreover, *KLF1* and *KLF17* were undetectable in HCC tissues (**Figure S1**). To preliminarily explore the function of the upregulated *KLFs* in HCC malignant behavior, we individually knocked down the four *KLF*s in MHCC97H cells and performed *in vitro* assays. Notably, *KLF7* exhibited the largest fold change among the elevated *KLFs* and KLF7 downregulation significantly inhibited HCC cell migration, invasion, and proliferation (**Figure S2**). Based on these findings, we focused on KLF7 in further studies.

**Figure 1:**
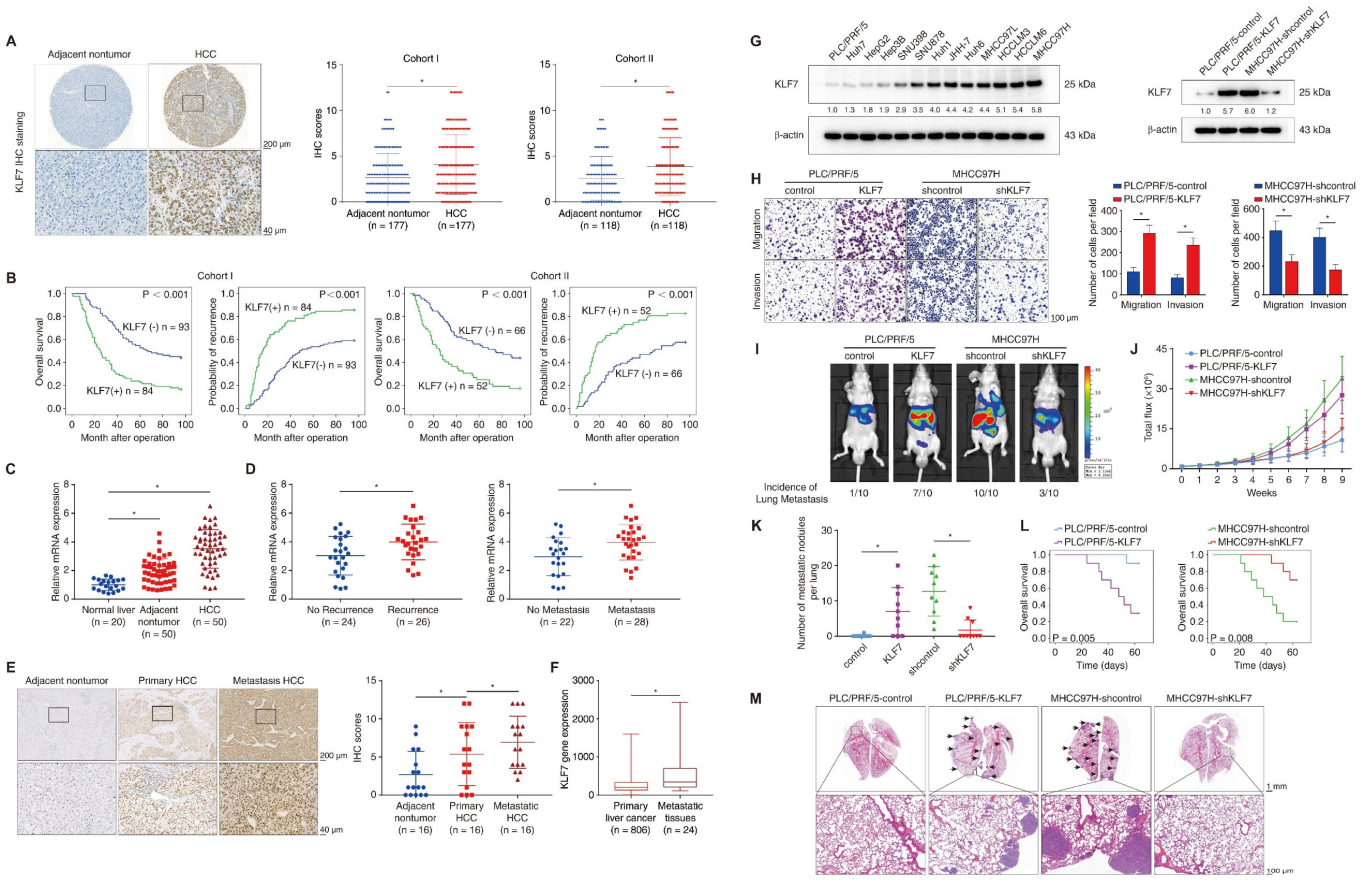
** KLF7 overexpression indicates poor prognosis in HCC patients and promotes HCC metastasis.** (A) Representative IHC images and IHC scores of KLF7 staining in para-cancerous nontumor specimens and HCC specimens in two independent microarrays. (B) Kaplan-Meier analysis of the correlation of KLF7 expression with overall survival and recurrence in two independent HCC cohorts. (C) Relative *KLF7* mRNA expression in 20 normal liver samples and 50 pairs of para-cancer nontumor and HCC tissues. (D) The mRNA expression of *KLF7* in primary HCC samples from patients without or with recurrence (left) and metastasis (right). (E) Representative IHC pictures and IHC scores of KLF7 staining in adjacent nontumorous samples, HCC samples, and matched metastatic HCC samples. (F) The gene expression of KLF7 in primary live tumors (n = 806) and liver cancer metastatic tissues (n = 24) was analyzed. Data from the TNMplot database (https://tnmplot.com/analysis/). (G) The protein levels of KLF7 in human HCC cell lines (left). KLF7 upregulation and knockdown in indicated HCC cells after lentiviral transfection were confirmed by immunoblotting (right). (H) The motility of indicated HCC cell lines with KLF7 expression changes were detected by transwell assays. (I-M) Effect of KLF7 overexpression on HCC metastasis were evaluated by orthotopic HCC models. (I) Representative bioluminescent images of hepatic tumors and incidence of pulmonary metastases. (J) Dynamic intensity of bioluminescent signals of liver tumors. (K) Number of lung metastatic nodules. (L) Overall survival time of nude mice. (M) Representative H&E staining of lung tissues. *P < 0.05. Abbreviations: IHC, Immunohistochemical; H&E, hematoxylin and eosin.

We next profiled the protein levels of KLF7 in two independent HCC cohorts using immunohistochemical (IHC) staining. KLF7 was predominantly localized in the nucleus and showed markedly elevated intensity in HCC tissues compared to para-cancer tissues (**Figure 1A**). In our HCC cohorts, KLF7-positive patients showed poorer OS and higher relapse probabilities than KLF7-negative patients (**Figure 1B**). Moreover, KLF7-positive patients exhibited a higher incidence of absent tumor encapsulation, microvascular invasion, poor tumor differentiation, and advanced tumor-node-metastasis (TNM) stage (**Table S1**). Positive KLF7 expression was also an independent predictor of poor OS and high recurrence in HCC patients, according to multivariate analysis (**Table S2**). HCC samples had higher *KLF7* mRNA expression than adjacent non-tumor samples and normal livers (**Figure 1C**), and HCC patients who had ever undergone recurrence or metastasis displayed elevated *KLF7* expression compared to those without recurrence or metastasis (**Figure 1D**). The protein levels of KLF7 were also markedly higher in metastatic HCC tissues than in primary tumors (**Figure 1E**), which was supported by online transcriptome data (**Figure 1F**). These findings collectively suggested that KLF7 elevation may be a key driver of HCC progression, especially in HCC metastasis.

### KLF7 overexpression promotes HCC metastasis

KLF7 expression was examined in different HCC cell lines (**Figure 1G**, left). PLC/PRF/5 cells with low endogenous KLF7 expression and low metastatic capabilities and MHCC97H cells with high endogenous KLF7 expression and high metastatic capabilities were chosen for gain and loss-of-function studies (**Figure 1G,** right). KLF7 upregulation enhanced the migration and invasion of PLC/PRF/5 cells, whereas KLF7 knockdown impaired the mobility of MHCC97H cells (**Figure 1H**). KLF7 overexpression also decreased epithelial marker E-cadherin expression and increased mesenchymal marker vimentin expression in HCC cells, and vice versa (**Figure S3**). Orthotopic HCC models further revealed that mice implanted with PLC/PRF/5-KLF7 cells exhibited higher pulmonary metastasis and shorter OS than those in the control group. In contrast, mice from the MHCC97H-shKLF7 group showed lower pulmonary metastasis rates and prolonged OS than those from the MHCC97H-shcontrol group (**Figure 1I-M**). These observations demonstrated that KLF7 promotes HCC metastasis.

### KLF7 upregulates TLR4 and PTK2 in HCC cells

**Figure 2:**
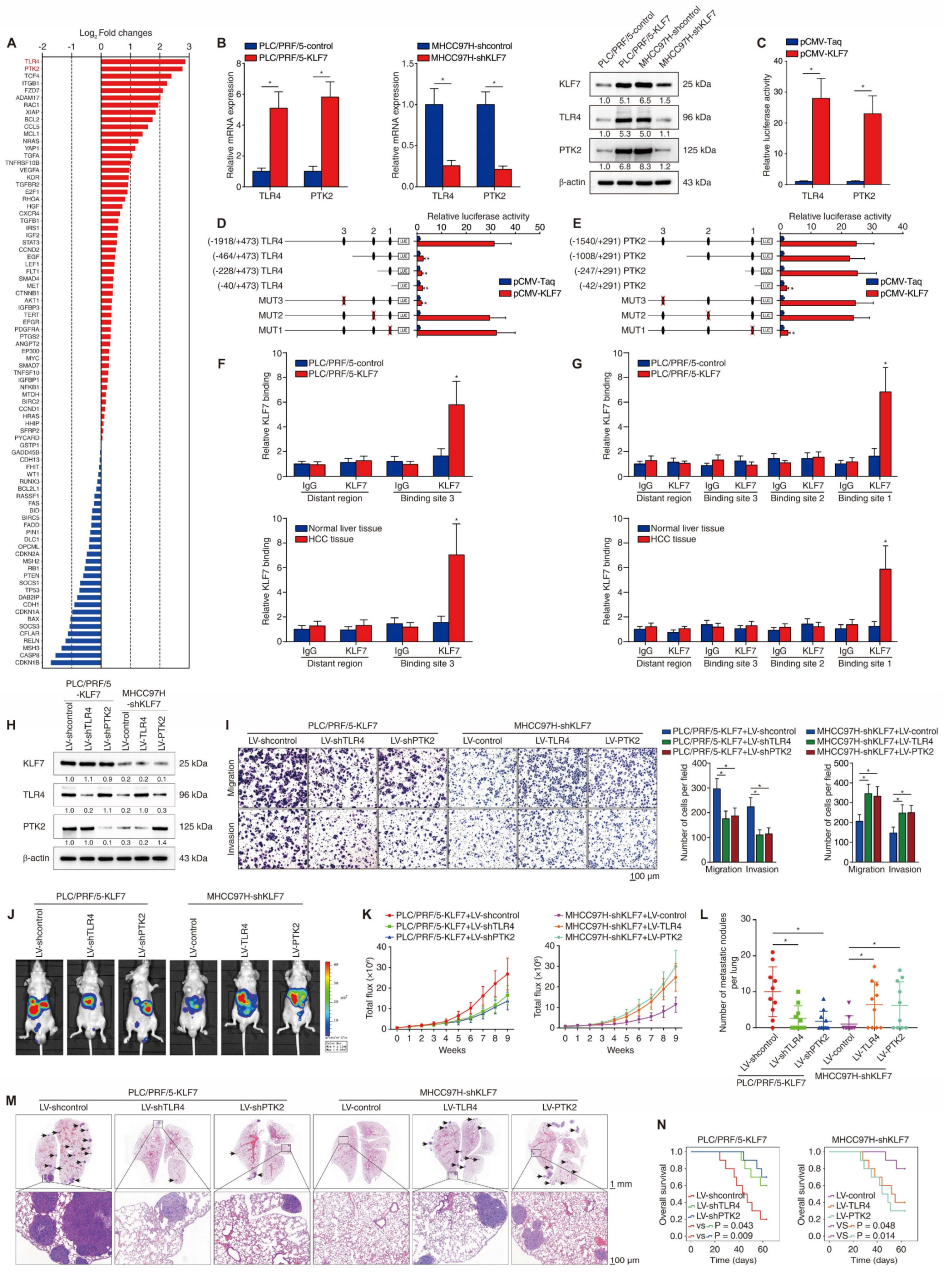
** KLF7 fosters HCC metastasis through transcriptionally upregulating TLR4 and PTK2 expression.** (A) Differentially-expressed genes between PLC/PRF/5-KLF7 cells and PLC/PRF/5-control cells were detected using A human liver cancer PCR array. (B) The mRNA and protein levels of TLR4 and PTK2 in PLC/PRF/5 cells with KLF7 overexpression or in MHCC97H cells with KLF7 knockdown. (C) Relative luciferase activities of *TLR4* and *PTK2* promotor reporter plasmids in PLC/PRF/5 cells co-transfected with pCMV-KLF7 or pCMV-Taq. (D-E) Serially truncated/mutated *TLR4* or *PTK2* promotor constructs were co-transfected with pCMV-KLF7 into PLC/PRF/5 cells for testing luciferase activities. (F-G) ChIP assays showed KLF7 directly bound to the *TLR4* and *PTK2* promoters in HCC cells and HCC specimens. (H) TLR4 and PTK2 knockdown in KLF7-overexpressing PLC/PRF/5 cells and upregulation in MHCC97H cells with KLF7 knockdown were confirmed by western blot. (I) The migratory and invasive capacities of the indicated HCC cell lines were evaluated by transwell assays. (J-N) *In vivo* metastatic experiments revealed that TLR4 and PTK2 upregulation was essential for KLF7-fostered HCC metastasis. (J) Representative bioluminescent pictures of liver tumors and rate of lung metastasis. (K) Bioluminescent signals of liver tumors were dynamically monitored. (L) Number of lung-colonizing nodules. (M) Typical H&E pictures of metastatic lung nodules. (N) Overall survival of different groups. *P < 0.05.

To investigate the molecular mechanism underlying KLF7-mediated HCC metastasis, we compared transcriptome changes between PLC/PRF/5-control and PLC/PRF/5-KLF7 cells using a Human Liver cancer RT^2^ Profiler PCR Array. Fourteen of the eighty-four genes were upregulated (fold change > 2.0) after KLF7 overexpression (**Figure 2A**, **Table S3**). Of particular interest were TLR4 and PTK2, which were ranked as the two most upregulated genes. Considering that both TLR4 and PTK2 are upregulated in HCC tissues and play critical roles in promoting HCC metastasis 16, 17, we further explored whether they are downstream effectors in KLF7-mediated HCC metastasis. KLF7 overexpression upregulated TLR4 and PTK2 expression, whereas KLF7 knockdown decreased TLR4 and PTK2 levels in HCC cells (**Figure 2B**). The luciferase activities of *TLR4* and *PTK2* promoters were also increased in KLF7-overexpressing cells, indicating that *TLR4* and *PTK2* were transcriptionally activated by KLF7 in HCC cells (**Figure 2C**).

Through sequence analysis, we identified multiple putative KLF7-binding motifs in both the TLR4 promoter (n = 3) and the PTK2 promoter (n =3) (**Figure S4, S5**). Serially truncated or mutated *TLR4* promoter reporter plasmids were constructed to explore the functions of these motifs in the transcriptional regulation of *TLR4*. Deletion of the fragment from -1918 to -464 base pairs (bp) significantly impaired KLF7-induced *TLR4* promoter activation. Consistently, mutation of putative KLF7-binding motif 3 within this region also abolished KLF7-activated *TLR4* promoter activity (**Figure 2D**). Likewise, we used the same approach to characterize KLF7-dependent *cis* elements in the *PTK2* promoter and identified that the putative KLF7-binding motif 1 in the -247bp ~ -42bp fragment was required for KLF7-induced *PTK2* promoter transactivation (**Figure 2E**). A chromatin immunoprecipitation (ChIP) assay further validated that KLF7 directly bound to the *TLR4* and *PTK2* promoters in both HCC cells and human HCC samples (**Figure 2F-G**). These results demonstrated that KLF7 transcriptionally upregulates TLR4 and PTK2 in human HCC cells.

### TLR4 and PTK2 are essential for KLF7-provoked HCC metastasis

Previous studies have demonstrated that both TLR4 and PTK2 play important roles in promoting HCC metastasis 16, 17. To investigate whether TLR4 and PTK2 function in KLF7-mediated HCC metastasis, we downregulated TLR4 or PTK2 expression in KLF7-overexpressing PLC/PRF/5 cells and upregulated TLR4 or PTK2 in MHCC97H cells with KLF7 knockdown (**Figure 2H**). TLR4 or PTK2 knockdown attenuated the migration and invasion of PLC/PRF/5-KLF7 cells, whereas TLR4 or PTK2 upregulation largely rescued the mobility defects of MHCC97H-shKLF7 cells (**Figure 2I**).* In vivo* metastatic assays demonstrated that TLR4 or PTK2 knockdown in PLC/PRF/5-KLF7 cells dramatically reduced pulmonary metastatic loads while extending the OS time of nude mice. Reciprocally, upregulation of TLR4 or PTK2 rescued the decline in pulmonary metastasis and reduced OS time in mice implanted with MHCC97H-shKLF7 cells (**Figure 2J-N**). Additionally, the orthotopic HCC models showed that the impact of knockdown or overexpression of TLR4 or PTK2 on HCC metastasis was less significant in the KLF7 non-manipulated state than in the KLF7 manipulated state, further demonstrating that the TLR4 and PTK2 effects are downstream of KLF7 (**Figure S6**). These findings indicated that TLR4 and PTK2 are essential downstream effectors of KLF7-mediated HCC metastasis.

### KLF7 expression positively correlated with TLR4 and PTK2 expression in human HCC specimens

**Figure 3:**
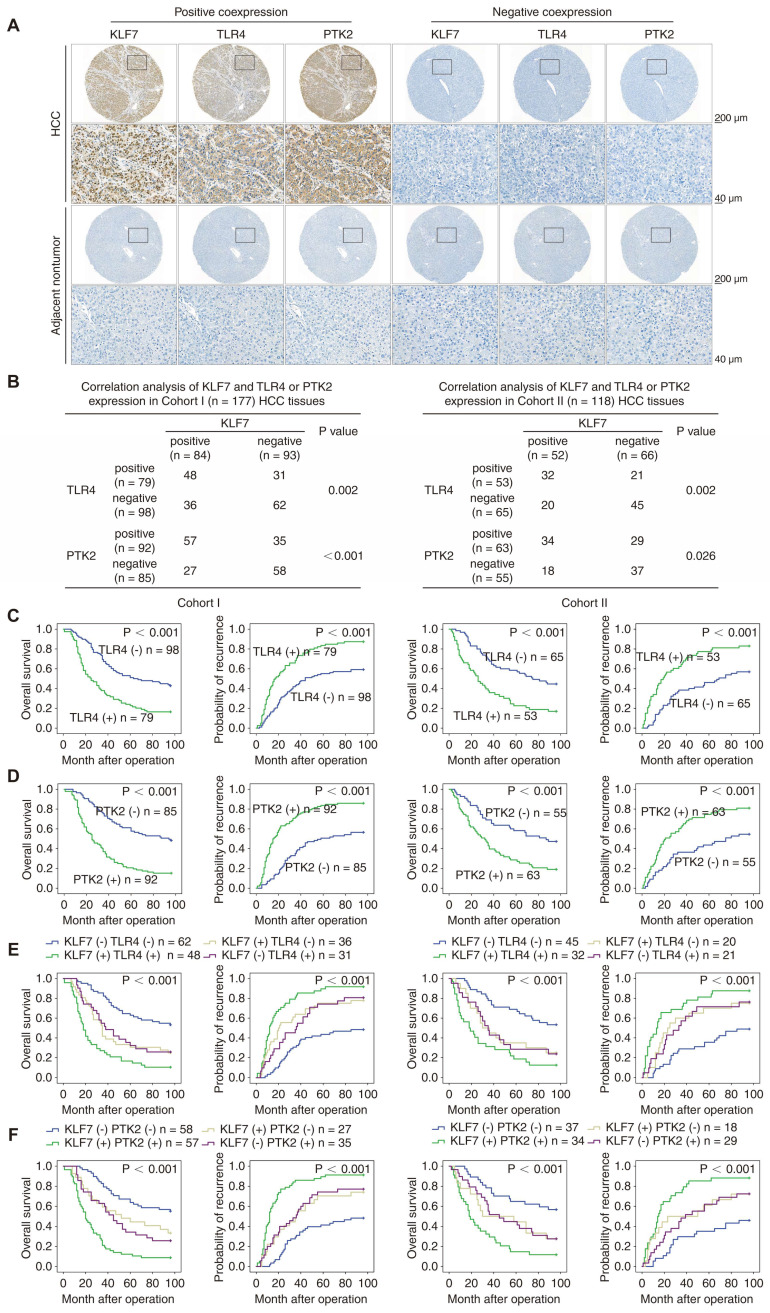
** KLF7 expression is positively correlated with TLR4 and PTK2 expression in human HCC.** (A) Representative IHC images of KLF7, TLR4 and PTK2 staining in adjacent nontumorous and HCC tissues. (B) The correlations between KLF7 expression and TLR4 or PTK2 expression in two HCC cohorts. (C, D) The prognostic significance of TLR4 or PTK2 expression on overall survival and recurrence rates in two HCC cohorts. (E, F) The correlation of KLF7/TLR4 or KLF7/PTK2 co-expression with overall survival and recurrence rates in two HCC cohorts. *P < 0.05.

IHC staining was performed to evaluate the clinical relevance of KLF7, TLR4, and PTK2 in HCC cohorts. Representative IHC staining of KLF7, TLR4, and PTK2 in HCC tissues and adjacent non-tumor tissues is presented (**Figure 3A**). Correlation analysis indicated that KLF7 expression was positively associated with TLR4 and PTK2 expression in both cohorts (**Figure 3B**). Positive TLR4 or PTK2 expression was accompanied by incomplete tumor encapsulation, microvascular invasion, poor differentiation, advanced TNM stage, poor OS, and high recurrence rates in our cohorts (**Figure 3C-D, Table S4, S5**). Notably, the subgroups of HCC patients co-expressing KLF7/TLR4 or KLF7/PTK2 displayed the worst prognosis in our cohorts (**Figure 3E**-**F**). Online bioinformatics analysis further validated these clinical observations. In TCGA LIHC samples, positive correlations were observed between *KLF7* and *TLR4* or *PTK2* expression, and HCC patients with high levels of *KLF7*/*TLR4* or *KLF7*/*PTK2* co-expression exhibited an obviously poorer OS than patients with low levels (**Figure S7A -B**).

To evaluate the pro-metastatic properties of KLF7, TLR4, and PTK2 in clinical samples, we compared their expression levels in 20 pairs of primary and metastatic HCC tissues. KLF7, TLR4, and PTK2 levels were markedly elevated in metastatic HCC tissues compared to those in primary HCC and adjacent non-neoplastic tissues (**Figure S8**). Correspondingly, by employing gene signature analysis in online LIHC databases, we found that the mean expression of the gene set composed of *KLF7*, *TLR4* and *PTK2* was elevated in liver cancer tissues compared to normal livers, and further increased in liver cancer metastatic tissues (**Figure S9A**). High levels of this gene set predicted much poorer survival in HCC samples (**Figure S9B**). These clinical observations collectively supported the conclusion that KLF7 promotes HCC metastasis by upregulating TLR4 and PTK2.

### Extracellular HMGB1 upregulates KLF7 expression through the TLR4/RAGE-PI3K-AKT-NF-κB signaling pathway

**Figure 4:**
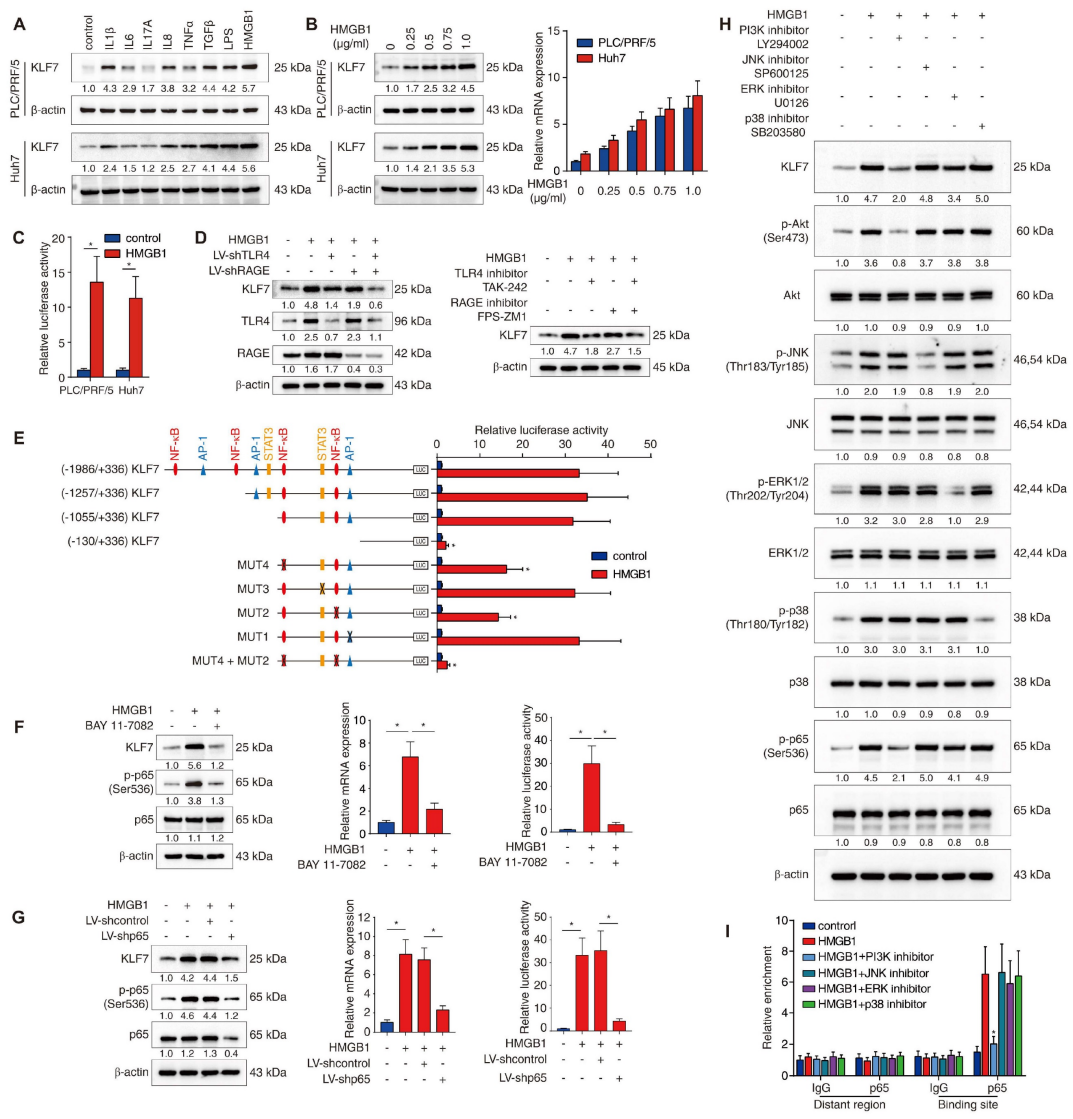
** HMGB1 induces KLF7 expression through the TLR4/RAGE-PI3K-AKT-NF-κB signaling pathway.** (A) PLC/PRF/5 and Huh7 cells were incubated with a panel of inflammatory factors for 24 hours, and KLF7 expression was then examined. (B) Western blotting and real-time PCR analysis of KLF7 expression in PLC/PRF/5 and Huh7 cells after HMGB1 treatment at a series of gradient doses for 24 hours. (C) The luciferase reporter activity of the* KLF7* promoter was measured in PLC/PRF/5 and Huh7 cells treated with HMGB1. (D) PLC/PRF/5 cells were transfected with LV-shTLR4, LV-shRAGE or a combination before HMGB1 treatment, KLF7, TLR4 and RAGE expression were then detected (left). TLR4 and RAGE inhibitors were applied in PLC/PRF/5 cells under HMGB1 treatment, and KLF7 expression was then examined (right). (E) PLC/PRF/5 cells were transfected with serially truncated or mutated *KLF7* promoter constructs and treated with or without HMGB1. Luciferase reporter activities were then detected. (F-G) PLC/PRF/5 cells were treated with NF-κB inhibitor BAY 11-7082 (F) or transfected with LV-shp65 (G) before HMGB1 stimulation. KLF7 expression and *KLF7* promoter activity were measured by western blotting, real-time PCR and luciferase reporter assays. (H) PLC/PRF/5 cells were precultured with inhibitors specific to PI3K, JNK, ERK and p38 before HMGB1 treatment. KLF7 expression as well as the levels of phosphorylated and total AKT, JNK, ERK, p38 and p65 were then detected. (I) A ChIP assay was conducted to examine the relative enrichment of p65 on the *KLF7* promoter when PLC/PRF/5 cells were treated with HMGB1 and inhibitors of PI3K, JNK, ERK and p38.

The upstream mechanism of KLF7 dysregulation in HCC cells still remains unclear. Chronic inflammation is a recognized hallmark of HCC and contributes to HCC metastasis 18. Inflammatory factors, including cytokines, damage-associated molecular patterns (DAMPs) and gut-derived pathogen-associated molecular patterns (PAMPs), are vital pro-metastatic signals in the HCC tumor microenvironment (TME) that can aberrantly activate pro-metastatic genes and pathways within HCC cells 18, 19. To determine whether proinflammatory factors triggered KLF7 overexpression in HCC cells, PLC/PRF/5 and Huh7 cells with low endogenous KLF7 expression were stimulated with a panel of inflammatory factors (IL-1β, IL-6, IL-17A, IL-8, TNF-α, TGF-β, LPS and HMGB1) enriched under chronic liver inflammation 19, 20. In both cell lines, HMGB1 was the most powerful inducer of KLF7 overexpression among all factors (**Figure 4A**). HMGB1 treatment induced KLF7 expression in a dose-dependent manner and significantly enhanced luciferase activity of *KLF7* promoter (**Figure 4B**-**C**). These observations suggested that extracellular HMGB1 upregulates KLF7 in HCC cells.

TLR4 and RAGE function as primary HMGB1 receptors and participate in HMGB1-mediated HCC metastasis 15, 21. To explore which receptor is required for HMGB1-induced KLF7 expression, PLC/PRF/5 cells were transfected with TLR4 knockdown, RAGE knockdown, or combined knockdown before HMGB1 treatment. Combined silencing of TLR4 and RAGE dramatically abolished HMGB1-induced KLF7 expression, whereas a single knockdown showed only a modest inhibitory effect (**Figure 4D**, left). Consistently, the combined blockade of TLR4 and RAGE showed more potent inhibition than a single drug treatment (**Figure 4D**, right). These data proved that both TLR4 and RAGE are required for HMGB1-induced KLF7 upregulation.

To identify the *cis*-regulatory elements responsible for HMGB1-dependent KLF7 expression, we analyzed the *KLF7* promoter and discovered multiple putative binding motifs of transcription factors downstream of TLR4/RAGE signaling (**Figure S10**). Next, we generated various luciferase reporter constructs containing truncated or mutated *KLF7* promoters and transfected them into PLC/PRF/5 cells. The absence of the -1055bp ~ -130bp region significantly decreased HMGB1-dependent *KLF7* promoter activity (**Figure 4E**). Co-mutation of the two NF-κB binding sites within this region largely impaired HMGB1-dependent *KLF7* promoter activity, whereas disruption of binding sites of other transcription factors had little effect (**Figure 4E**). Subsequently, PLC/PRF/5 cells were treated with p65 knockdown or pretreated with an NF-κB inhibitor prior HMGB1 administration. These operations inhibited NF-κB phosphorylation and reduced HMGB1-induced KLF7 overexpression and HMGB1-dependent *KLF7* promoter activity (**Figure 4F**-**G**). TLR4/RAGE signaling activates intracellular PI3K, JNK, ERK, and p38 pathways 22, 23. To identify which signaling pathway mediated HMGB1-dependent NF-κB activation, PLC/PRF/5 cells were treated with signaling-specific inhibitors. The PI3K inhibitor largely abolished, while the ERK inhibitor slightly lowered HMGB1-induced NF-κB phosphorylation and KLF7 overexpression (**Figure 4H**). Furthermore, a ChIP assay validated that PI3K inhibitor treatment significantly mitigated the binding of NF-κB to the *KLF7* promoter, while JNK, ERK, and P38 inhibitors had no such obvious effect (**Figure 4I**). Above all, these findings indicated that extracellular HMGB1 upregulates KLF7 expression through the TLR4/RAGE-PI3K-AKT-NF-κB signaling pathway in HCC cells.

### KLF7 is crucial for HMGB1-induced HCC metastasis

**Figure 5:**
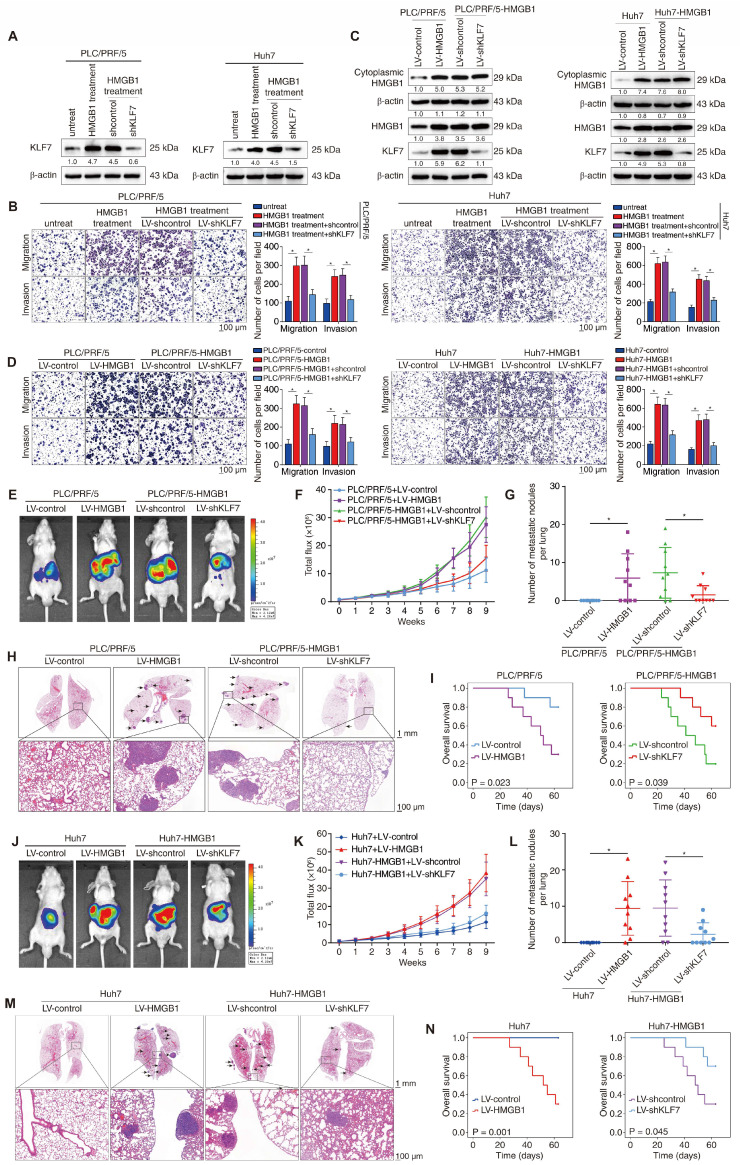
** KLF7 is essential for HMGB1-induced HCC metastasis.** (A) PLC/PRF/5 cells and Huh7 with or without KLF7 knockdown were treated with HMGB1, and KLF7 expression was examined by Western blotting. (B) Transwell analysis of the mobility of indicated PLC/PRF/5 cells and Huh7 cells. (C) Lentivirus infection was used to upregulate HMGB1 in PLC/PRF/5 and Huh7 cells, and silence KLF7 in HMGB1-overexpressing PLC/PRF/5 and Huh7 cells. The protein levels of cytoplasmic HMGB1, total HMGB1 and KLF7 were examined. (D) The migration and invasion of PLC/PRF/5-HMGB1 cells and Huh7-HMGB1 cells with or without KLF7 knockdown were assessed by transwell assays. (E-I) Orthotopic HCC models shown that KLF7 knockdown abrogated HMGB1-dependent HCC metastasis. The nude mice were injected with the indicated PLC/PRF/5 cells in the livers. (E) Typical bioluminescent images of hepatic tumors in different groups. (F) Bioluminescent signals of liver tumors. (G) Number of metastatic nodules in lung. (H) Representative H&E images of metastases in lung tissues. (I) Overall survival time of nude mice. (J-N) Orthotopic HCC models shown that KLF7 downregulation inhibited HMGB1-mediated HCC metastasis. The nude mice were injected with the indicated Huh7cells in the livers. (J) Typical bioluminescent images of hepatic tumors in different groups. (K) Bioluminescent signals of liver tumors. (L) Number of metastatic nodules in lung. (M) Representative H&E images of metastases in lung tissues. (N) Overall survival time of nude mice. *P < 0.05.

The pivotal role of HMGB1 in promoting HCC metastasis has been identified 15. Considering that KLF7 is induced by HMGB1 and facilitates HCC metastasis, we investigated its role in HMGB1-mediated HCC metastasis. HMGB1 was used to stimulate PLC/PRF/5 and Huh7 cells, with or without KLF7 knockdown (**Figure 5A**). HMGB1 treatment markedly enhanced the migration and invasion of PLC/PRF/5 and Huh7 cells, whereas KLF7 downregulation largely reversed these changes (**Figure 5B**). Consistently, HMGB1 upregulation augmented the migration and invasion of PLC/PRF/5 and Huh7 cells, whereas KLF7 knockdown mostly abolished HMGB1-enhanced migration and invasion of HCC cells (**Figure 5C-D**). Moreover, nude mice implanted with PLC/PRF/5-HMGB1 or Huh7-HMGB1 cells exhibited higher pulmonary metastasis and poorer OS than their control groups, whereas KLF7 knockdown significantly reversed these HMGB1-dependent phenotypes (**Figure 5E-I, Figure 5J-5N**). These data indicated that KLF7 elevation is crucial for HMGB1-induced HCC metastasis.

### KLF7 expression was positively associated with cytoplasmic HMGB1 and phosphorylated NF-κB p65 levels in human HCC specimens

**Figure 6:**
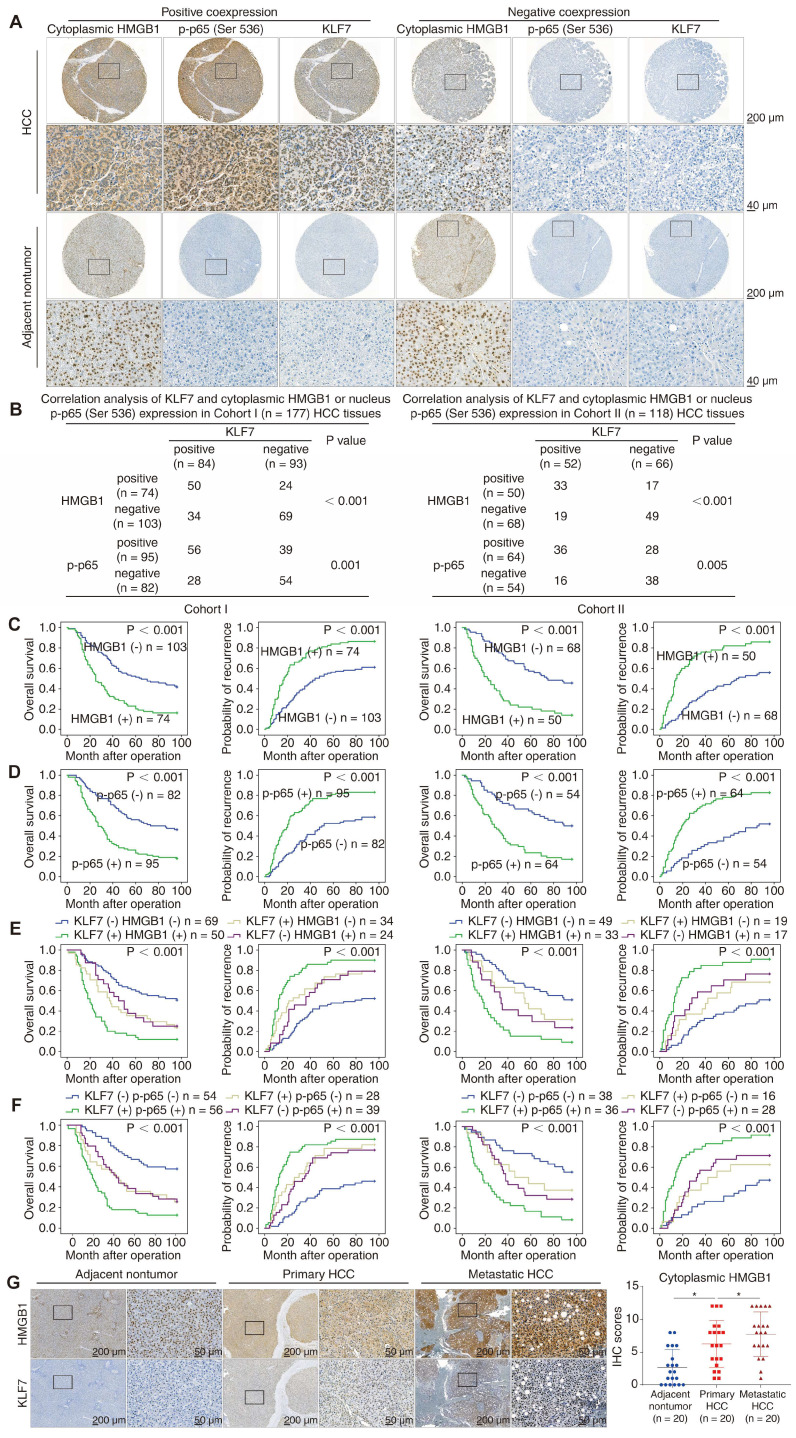
** KLF7 expression is positively correlated with the expression levels of cytoplasmic HMGB1 and nucleus p-p65 in human HCC tissues.** (A) Representative IHC staining of HMGB1, p-p65(Ser536) and KLF7 expression in HCC tissues and para-cancer nontumor specimens. (B) Correlation analysis of KLF7 expression and cytoplasmic HMGB1 or nucleus p-p65 (Ser536) expression in two independent HCC cohorts. (C-D) Overall survival and recurrence rates of HCC patients with positive or negative cytoplasmic HMGB1 (C) or p-p65 (D) in two independent cohorts. (E-F) The correlation of KLF7/cytoplasmic HMGB1 (E) or KLF7/p-p65 (F) co-expression and overall survival and recurrence rates in our HCC cohorts. (G) Representative IHC staining of HMGB1 and KLF7 expression in para-cancerous nontumor samples, HCC specimens, and metastatic HCC tissues. IHC scores of and HMGB1 were presented. *P < 0.05.

To confirm the clinical significance of HMGB1, p-p65 (Ser536), and KLF7 in HCC tissues, we profiled their expression using IHC staining in our HCC cohorts. Representative IHC images of HMGB1, p-p65(Ser536), and KLF7 expression are shown (**Figure 6A**). While para-cancerous non-tumor tissues always displayed nuclear HMGB1 immunoreactivity, positive cytoplasmic expression of HMGB1 was detected in nearly half of the analyzed HCC tissues. Compelling data demonstrated that HMGB1 can translocate from the nuclear to the cytoplasm and then secrete into the extracellular context as a danger alarm in inflammatory/stress hepatocytes 11. Thus, cytoplasmic HMGB1 levels can be used as a sign to reflect HMGB1 secretion levels in HCC tissues 24, 25. In our HCC cohorts, KLF7 expression positively correlated with cytoplasmic HMGB1 and nucleus p-p65 levels (**Figure 6B**). HCC patients with cytoplasmic HMGB1 or nucleus p-p65 upregulation displayed poor prognosis and aggressive pathological parameters (**Figure 6C**-**D, Tables S6, S7**). Furthermore, patients with either HMGB1/KLF7 or p-p65/KLF7 positive co-expression had the poorest prognosis in our cohorts (**Figure 6E**-**F**). Data from the TCGA LIHC cohort also confirmed that *KLF7* expression was positively correlated with *HMGB1* and *RELA* expression in HCC samples, and HCC patients highly co-expressing *HMGB1*/*KLF7* or *RELA*/*KLF7* had significantly poorer OS, especially in Asian subpopulations (**Figure S7C-D**). Furthermore, we detected the expression levels of HMGB1 and KLF7 by IHC staining in 20 pairs of primary and metastatic HCC tissues. The staining of HMGB1 in metastatic HCC tissues was primarily localized in the cytoplasm and was more intense than in primary HCC tissues, which was in accordance with the KLF7 expression pattern (**Figure 6G**).

### Hepatocyte-specific knockout of *Klf7* impedes DEN/CCl_4_-induced HCC development and metastasis

**Figure 7:**
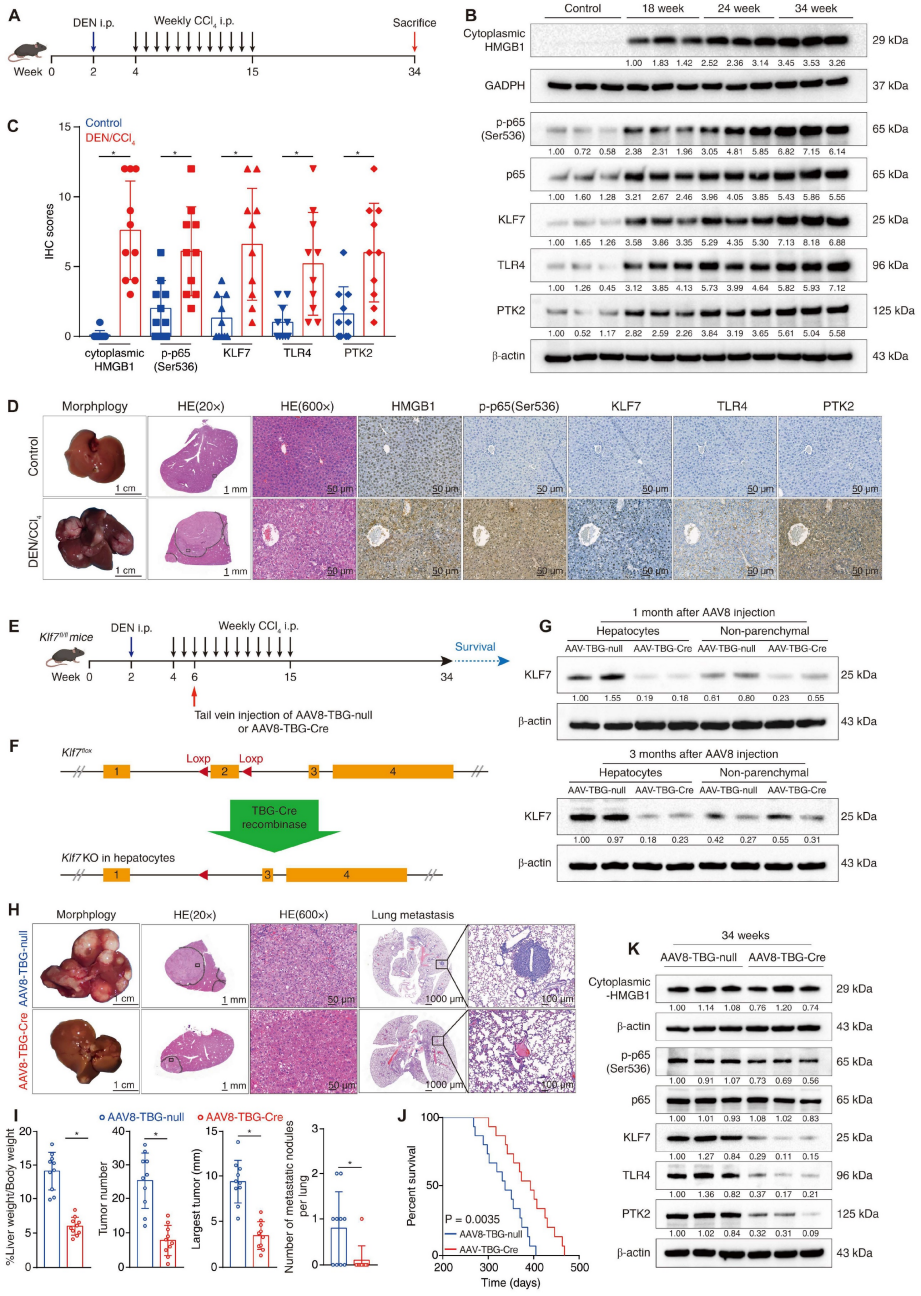
** Hepatocyte-specific knockout of *Klf7* impedes DEN/CCl_4_-induced HCC development and metastasis.** (A) Schematic diagram of DEN/CCl_4_-induced HCC model. (B) The levels of cytoplasmic HMGB1, p-p65, p65, KLF7, TLR4, and PTK2 in the livers of control mice or in mice livers from the DEN/CCl4-treated group at the indicated time points were detected by immunostaining. (C-D) IHC scores of the levels of HMGB1, p-p65, KLF7, TLR4, and PTK2 in mice livers from the control group and in HCC tissues from the DEN/CCl_4_ group at 34 weeks (C). Representative IHC staining of each group was shown (D). (E) The schematic showed the time point of AAV8-TBG-null or AAV8-TBG-Cre injection during DEN/CCl_4_ treatment. (F) The schematic showed the mechanism by which *Klf7* was knockout in hepatocytes by TBG-Cre recombinase in the *Klf7*^fl/fl^ mice. (G) Expression of KLF7 in hepatocytes and non-parenchymal cells of AAV8-TBG-null and AAV8-TBG-Cre groups at the indicated time points after AAV8 injection. (H) Representative pictures of whole-liver morphology, H&E of liver sections, and H&E of lung metastases from the AAV8-TBG-null and AAV8-TBG-Cre groups at 34 weeks. (I) Liver weight/body weight ratio, tumor number, largest tumor size, and the number of pulmonary metastatic nodules in each group were statically analyzed at 34 weeks (n = 10 per group). (J) 15 mice in each group were raised until natural death for survival analysis. Survival rates of *Klf7*^fl/fl^ mice treated with AAV8-TBG-null or AAV8-TBG-Cre were shown (n = 15 per group). (K) The protein levels of cytoplasmic HMGB1, p-p65, KLF7, TLR4, and PTK2 in the liver tumors from the AAV8-TBG-null group and the AAV8-TBG-Cre group at 34 weeks were examined by immunostaining. *P < 0.05.

The DEN/CCl_4_-induced HCC model can imitate the process of hepatic inflammation-cirrhosis-HCC sequence and is suitable for studying inflammation-mediated HCC progression and metastasis 26. Previous studies have reported that cytoplasmic translocation and secretion of HMGB1, as well as phosphorylation of NF-κB p65, were markedly upregulated in mouse livers following chronic diethyl nitrosamine (DEN) or carbon tetrachloride (CCl_4_) treatment 13, 27, 28, we thus wondered whether the HMGB1-p-p65-KLF7-TLR4/PTK2 pathway was activated in the DEN/CCl_4_-induced HCC model. By performing immunoblotting at different time points during DEN/CCl_4_ treatment, we observed that the cytoplasmic translocation of HMGB1 and the levels of p-p65, KLF7, TLR4 and PTK2 were gradually upregulated during HCC initiation and progression. (**Figure 7B**). IHC assays further confirmed that the HMGB1-p-p65-KLF7-TLR4/PTK2 pathway was more highly activated in DEN/CCl_4_-induced HCC tissues than in normal livers (**Figure 7C**-**D**).

To explore the role of KLF7 in HMGB1-mediated HCC development and metastasis, we performed hepatocyte-specific knockout of *Klf7* in *Klf7*^fl/fl^ mice via tail vein injection of AAV8-TBG-Cre vectors in the early stage of DEN/CCl_4_ treatment (**Figure 7E**-**F**). The effectiveness of *Klf7* knockout in the hepatocytes of *Klf7*^fl/fl^ mice was verified using western blotting (**Figure 7G**). Surprisingly, mice in the *Klf7* knockout group developed smaller hepatic tumors with fewer nodules and less pulmonary metastasis, while exhibiting a much longer survival time (**Figure 7H-J**). Hepatic tumors from the *Klf7* knockout group also showed low levels of KLF7, TLR4, and PTK2 (**Figure 7K**). These data proved that DEN/CCl_4_-induced HMGB1 release promotes HCC progression by activating the KLF7-TLR4/PTK2 axis in hepatocytes, and that hepatocyte-specific *Klf7* knockout significantly impedes HMGB1-induced HCC development and metastasis.

### Combined administration of TLR4 inhibitor TAK-242 and PTK2 inhibitor defactinib significantly alleviates KLF7-mediated HCC development and metastasis

**Figure 8:**
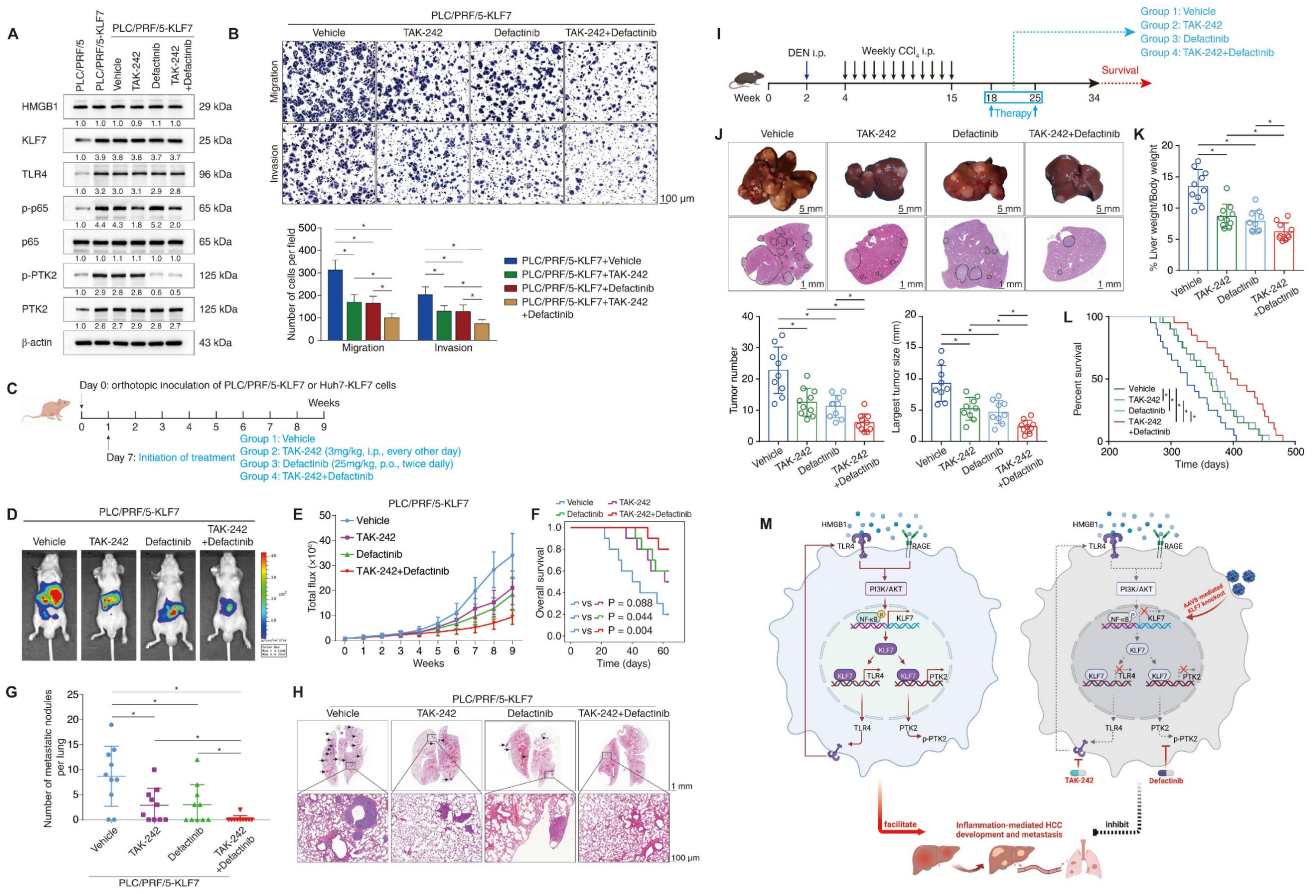
** Combined administration of TLR4 and PTK2 inhibitors suppresses KLF7-mediated HCC development and metastasis.** (A) PLC/PRF/5-KLF7 cells were incubated with vehicle, TAK-242, defactinib, or a combination of both, then the protein levels of HMGB1, KLF7, TLR4, p-p65, p65, p-PTK2 and PTK2 were detected. (B) Transwell analysis of the mobility of PLC/PRF/5-KLF7 cells treated with TAK-242 alone, defactinib alone, or a combination of both. (C) The diagram of drug treatment. Nude mice were transplanted with HCC cells and randomly divided into four groups. Then, vehicle, TAK-242, defactinib, or combined administration were applied to different groups, respectively. (D-H) Combined blockade of TLR4 and PTK2 alleviated KLF7-mediated HCC metastasis. (D) Representative bioluminescent pictures of liver tumors in nude mice. (E) Intensity of bioluminescent signals of liver tumors. (F) Overall survival of mice. (G)The amount of pulmonary metastatic nodules. (H) Representative pictures of H&E staining of lung tissues. (I) Schematic diagram of DEN/CCl_4_ treatment and drug regimens. Mice were randomly divided into four groups at 18 weeks. Then, vehicle, TAK-242, defactinib, or combined application was applied to each group, respectively. (J) Typical images of whole-liver morphology and H&E of liver sections from different groups at 34 weeks. (K) 10 mice in each group were sacrificed at 34 weeks for statistical analysis. The analysis of liver weight/body weight ratio, tumor number, and largest tumor size in each group were shown (n = 10 per group). (L) 20 mice in each group were raised until natural death for survival analysis. The survival rates of mice in different groups were shown (n = 20 per group). (M) A schematic diagram depicting the function of the HMGB1-KLF7-TLR4/PTK2 pathway in inflammation-mediated HCC development and metastasis. Inflammation-provoked HMGB1 release upregulates KLF7 expression via the TLR4/RAGE-PI3K-AKT-NF-κB pathway within HCC cells. Furthermore, KLF7 promotes HCC development and metastasis by transcriptionally upregulating TLR4 and PTK2 expression, forming an HMGB1-KLF7-TLR4 positive feedback loop. Hepatocyte-specific knockout of *Klf7* via AAV8 gene therapy, or combined administration of TLR4 inhibitor TAK-242 and PTK2 inhibitor defactinib significantly impedes HCC development and metastasis mediated by the HMGB1-KLF7 axis. *P < 0.05.

Given that TLR4 and PTK2 are downstream effectors of the HMGB1-KLF7 axis, we explored whether the pharmacological inhibition of TLR4 and PTK2 could impede KLF7-mediated HCC development and metastasis. The small-molecule TLR4 inhibitor TAK-242 and PTK2 inhibitor defactinib attracted our attention because of their verified safety and efficacy profiles 29, 30. PLC/PRF/5 and Huh7 cells stably overexpressing KLF7 were treated with TAK-242, defactinib, or a combination of both drugs. The efficacy of TAK-242 and defactinib was verified by decreased levels of p-p65 and p-PTK2, respectively (**Figure 8A, Figure S11A**). TAK-242 or defactinib alone partly while their combination potently inhibited the migration and invasion of PLC/PRF/5-KLF7 and Huh7-KLF7 cells (**Figure 8B**, **Figure S11B**). Consistently, the combination of TAK-242 and defactinib significantly alleviated pulmonary metastatic burden and extended OS time compared to the single-agent groups in nude mice implanted with PLC/PRF/5-KLF7 or Huh7-KLF7 cells (**Figure 8D-H, Figure S11C-G**). We examined the therapeutic effects of our combination strategy in the DEN/CCl_4_-induced HCC model. Combination therapy with TAK-242 and defactinib markedly suppressed HCC development and extended mouse survival time, while single-drug treatment showed moderate efficacy (**Figure 8I-L**).

## Discussion

Transcriptional factors are vital integrators of input oncogenic signals and represent the central nodes regulating tumor biology in HCC 31. Genomic and transcriptomic data on human HCC revealed that many transcription factors are dysregulated in aggressive HCC subtypes, suggesting a promising prospect for translating dysfunctional transcription factors into HCC stratification and therapy 31. Here, we identified KLF7 elevation as an independent prognostic predictor of HCC and highlighted its vital role in HCC progression and metastasis. As a transcription factor, KLF7 exerts cancer-promoting effects through transcriptional activation of oncogenic genes in human cancers 6, 8. Our study demonstrated that KLF7 transcriptionally upregulates TLR4 and PTK2 expression in HCC cells. TLR4 is a transmembrane pattern-recognition receptor that recognizes both PAMPs and DAMPs and senses danger signals 32. TLR4 is overexpressed in HCC tissues and predicts poor survival and early recurrence 16, 33. The TME of HCC is commonly enriched with a variety of TLR4 ligands such as gut-derived lipopolysaccharide (LPS), dying or stressed cell-released HMGB1/heat shock protein 70 (HSP70), and hepatitis B virus (HBV) antigens 32. These ligands persistently stimulate HCC cells overexpressing TLR4, ultimately leading to hyperactivation of TLR4 signaling. Activated TLR4 signaling subsequently induces hepatocarcinogenesis and promotes HCC growth, stemness, EMT and metastasis 16, 33, 34. PTK2, also known as focal adhesion kinase (FAK), is a non-receptor tyrosine kinase downstream of multiple extracellular signals such as growth factors, integrins, and cytokines 35. PTK2 is upregulated and hyperphosphorylated in multiple tumors and contributes to poor prognosis 35. In human HCC, PTK2 expression is elevated and serves as an independent prognostic factor 36. PTK2 overexpression significantly accelerates hepatocarcinogenesis and enhances HCC growth, invasion, metastasis, and self-renewal 17, 37-39. Thus, TLR4 and PTK2 are vital regulators of HCC progression and metastasis, respectively. Our study found that KLF7 promotes HCC progression and metastasis by transcriptionally upregulating TLR4 and PTK2. In HCC samples, KLF7 expression was positively correlated with TLR4 and PTK2 expression, and patients positively co-expressing KLF7/TLR4 or KLF7/PTK2 exhibited the worst prognosis.

As a typical inflammation-induced cancer, HCC generally has an inflammatory TME enriched with cytokines, PAMPs, and DAMPs, which are vital pro-tumorigenic and pro-metastatic signals for HCC cells 18. In this study, we found that inflammatory factors triggered KLF7 expression in HCC cells and identified HMGB1 as a potent inducer of KLF7 upregulation. HMGB1, a typical DAMP, can be translocated from the nucleus to the cytoplasm and subsequently secreted during cell injury, HBV X protein stimulation, hypoxia, sustained p53 activation, etc. 13, 24, 40, which leads to HMGB1 accumulation in the extracellular milieu of chronic liver diseases and HCC 11. Extracellular HMGB1 ulteriorly facilitates HCC initiation, growth, and metastasis by interacting with cell-surface TLR4/RAGE receptors and endosome-membrane TLR9 receptor 14, 15, 40. Immunostaining of HMGB1 in HCC specimens is primarily observed in the cytoplasm of carcinoma cells and is stronger than in para-tumors 41. High cytoplasmic HMGB1 levels are positively associated with poor prognosis and aggressive pathological parameters in HCC patients 25, 41. Therefore, extracellular HMGB1 is an inflammation-related oncogenic factor in human HCC. Our study revealed that extracellular HMGB1 induced KLF7 elevation in HCC cells through the TLR4/RAGE-PI3K-AKT-NFκB signaling pathway. Genetic depletion of KLF7 significantly inhibited HMGB1-mediated HCC progression and metastasis. In the HCC cohorts, KLF7 expression was positively associated with cytoplasmic HMGB1 intensity, and positive HMGB1/KLF7 co-occurrence predicted the worst prognosis. Moreover, high KLF7 expression predicted inferior OS in HMGB1-high HCC patients. In conclusion, our data identified a novel HMGB1-KLF7 axis involved in inflammation-induced HCC progression and metastasis.

To pharmacologically inhibit the HMGB1-KLF7 axis in HCC, we focused on small-molecule inhibitors targeting its downstream effectors, TLR4 and PTK2. TAK-242 (resatorvid) is a selective small-molecule TLR4 inhibitor that interferes with interactions between the TLR4 intracellular domain and its adaptor molecules 29. An attempt to use TAK-242 for treating human sepsis has reached phase Ⅲ trials, confirming the safety of this drug 29. Recently, TAK-242 alone or in combination with other drugs, has shown anti-tumor effects in preclinical models of colorectal cancer, breast cancer, glioma, and skin cancer 42-45. Defactinib (VS-6063) is an orally bioavailable PTK2 inhibitor that suppresses Y397 phosphorylation of PTK2 via an ATP-competitive mechanism 30. Several completed clinical trials on solid tumors have proven the tolerable safety profile and modest efficacy of defactinib 30. Moreover, defactinib monotherapy or co-administration with other agents has shown exciting therapeutic efficacy in preclinical models of ovarian cancer, PDAC, and EGFR-mutant non-small-cell lung cancer 46-48. This study found that the combined administration of TAK-242 and defactinib effectively inhibited KLF7-mediated HCC progression and metastasis, as well as prolonged OS in preclinical HCC mouse models. Gene signature analysis further revealed that the KLF7-TLR4/PTK2 axis is activated in HCC tissues and contributes to HCC metastasis and poor prognosis. These findings indicate an excellent application prospect for translating our combination strategy into treating the KLF7-positive HCC subpopulation.

In conclusion, extracellular HMGB1 induced KLF7 expression via the TLR4/RAGE-PI3K-AKT-NF-κB pathway in HCC cells. KLF7 was critical for HMGB1-mediated HCC progression and metastasis. The HMGB1-KLF7 axis facilitated HCC progression and metastasis by upregulating TLR4 and PTK2 expression, forming an HMGB1-KLF7-TLR4 positive feedback loop. Combined administration of the TLR4 inhibitor TAK-242 and PTK2 inhibitor defactinib or genetic ablation of KLF7 in hepatocytes significantly impeded the HMGB1-KLF7 axis-mediated HCC progression and metastasis, which represented promising therapeutic strategies for KLF7-positive HCC patients.

## Supplementary Material

Supplementary materials and methods, figures and tables.

## Abbreviations

HCC: hepatocellular carcinoma
KLF7: krüppel-like factor 7
TLR4: toll-like receptor 4
PTK2: protein tyrosine kinase 2
HMGB1: high mobility group box 1
RAGE: advanced glycosylation end-product specific receptor
RELA: RELA proto-oncogene, NF-κB subunit
DAMPs: damage-associated molecular patterns
PAMPs: pathogen-associated molecular patterns
AAV8: adeno-associated virus 8
H&E: Hematoxylin and eosin
IHC: immunohistochemistry
mRNA: messenger RNA
shRNA: short hairpin RNA
ChIP: chromatin immunoprecipitation analysis
BLI: bioluminescent imaging
TCGA: The Cancer Genome of Atlas
LIHC: liver hepatocellular carcinoma
OS: overall survival
RFS: relapse-free survival
DFS: disease-free survival
HR: hazard ratio
