# Supplementary material for: HMGB1-mediated elevation of KLF7 facilitates hepatocellular carcinoma progression and metastasis through upregulating TLR4 and PTK2
Source: Theranostics. 2023 Jul 14;13(12):4042–58. doi: 10.7150/thno.84388 (PMC10405848; doi:10.7150/thno.84388)
Supplement: Supplementary file 1 — Supplementary materials and methods, figures and tables. [file thnov13p4042s1.pdf]

Supplementary materials

Materials and Methods

Cell culture

HCC cells (Huh1, Huh6, Huh7, JHH-7, and SNU878) were purchased from the Institute of Biochemistry and Cell Biology, Chinese Academy of Science, China. Human HCC cells (HepG2, Hep3B, PLC/PRF/5, SNU398) were purchased from the American Type Culture Collection. Additional human HCC cells (MHCC97L, MHCC97H, HCCLM3, and HCCLM6) were kindly provided by Dr. Tang ZY (Liver Cancer Institute, Zhongshan Hospital, Fudan University, Shanghai, China). Cells were cultured in Dulbecco's Modified Eagle Medium (DMEM) or RPMI-1640 medium at 37 °C in a 5% CO_2 incubator. The medium was supplemented with 10% FBS, 100 μg/mL penicillin, and 100 μg/ mL streptomycin. These above cell lines were authenticated by short tandem repeats (STRs) DNA profiling. All cells were tested for mycoplasma contamination before use with the Universal Mycoplasma Detection Kit (ATCC 30-1012K) and were not contaminated by mycoplasma.

Patients and follow-up

This study was approved by the Ethics Committee of Tongji Medical College, and all patients provided full consent for the study. Cohort I included 177 adult patients with HCC who underwent curative resection between 2003 and 2005 at the Tongji Hospital of Tongji Medical College (Wuhan, China). Cohort II included 118 adult patients with HCC who underwent curative resection between 2006 and 2008 at the Tongji Hospital

of Tongji Medical College (Wuhan, China). A preoperative clinical diagnosis of HCC was based on the diagnostic criteria of the American Association for the Study of Liver Diseases. The inclusion criteria were as follows: (a) distinctive pathologic diagnosis; (b) no preoperative anticancer treatment or distant metastases; (c) curative liver resection; and (d) complete clinicopathologic and follow-up data. The differentiation statuses were graded according to the method of Edmondson and Steine. The pTNM classification for HCC was based on The American Joint Committee on Cancer/International Union Against Cancer staging system (6th edition, 2002). Follow-up data were summarized at the end of December 2013 (Cohort I) and December 2016 (Cohort II, range 4-96 months) respectively. The patients were evaluated every 2-3 months during the first 2 years and every 3-6 months thereafter. All follow-up examinations were performed by physicians who were blinded to the study. During each check-up, the patients were monitored for tumor recurrence by measuring the serum AFP levels and by performing abdominal ultrasound examinations. Computed tomography and/or magnetic resonance imaging examination was performed every 3-6 months, together with a chest radiographic examination. The diagnostic criteria for HCC recurrence were the same as the preoperative criteria. The time to recurrence and overall survival were the primary endpoints. The time to recurrence was calculated from the date of resection to the date of a diagnosis with tumor recurrence. The overall survival was calculated from the date of resection to the date of death or of the last follow-up.

In addition, 10 normal liver tissues and 25 pairs of adjacent nontumor and HCC

tissues preserved in liquid nitrogen were used for examining the mRNA levels of *KLF* family genes (Figure S1); 20 normal liver tissues and 50 pairs of HCC tissues and adjacent nontumor tissue samples preserved in liquid nitrogen were used to detect the mRNA expression of *KLF7* (Figure 1C); 16 pairs of fresh adjacent nontumor tissues, primary HCC tissues and matched metastatic HCC tissues were collected after surgical resection and were used for KLF7 IHC staining (Figure 1E); 20 pairs of fresh adjacent nontumor tissues, primary HCC tissues and matched metastatic HCC tissues were collected after surgical resection and were used to detect the expression profiles of HMGB1, KLF7, TLR4, and PTK2 (Figure 6G, Figure S8).

Plasmid construction

Plasmid construction was performed according to standard procedures. The primers were shown in Table S8. For example, the *KLF7* gene complete CDS construct, pCMV-KLF7, was generated by using cDNA from human PBMCs. It was generated with forward and reverse primers incorporating EcoRI and HindIII sites at the 5' and 3'-ends, respectively. The polymerase chain reaction (PCR) product was cloned into the EcoRI and HindIII sites of the pCMV-Tag2B vector. The *TLR4* promoter construct, (-1918/+473) *TLR4*, was generated from human genomic DNA. This construct corresponds to sequence from -1918 to +473 (relative to the transcriptional start site) of the 5'-flanking regions of human *TLR4* gene. It was generated with forward and reverse primers incorporating SacI and NheI sites at the 5' and 3'-ends, respectively. The polymerase chain reaction (PCR) product was cloned into the SacI and NheI sites

of the pGL3-Basic vector (Promega). The 5'-flanking deletion constructs of the *TLR4* promoter, (-464/+473) *TLR4*, (-228/+473) *TLR4*, (-40/+473) *TLR4* were similarly generated using the (-1918/+473) *TLR4* construct as the template. The KLF7 binding sites in the *TLR4* promoter were mutated using the QuikChange II Site-Directed Mutagenesis Kit (Stratagene). The constructs were confirmed by DNA sequencing. Other promoter constructs were cloned in the same manner.

Construction of lentivirus and stable cell lines

Lentiviral vectors encoding shRNAs were generated using PLKO.1-TRC (Addgene) and designated as LV-shKLF7, LV-shTLR4, LV-shPTK2, LV-shRAGE, LV-shp65, and LV-shcontrol. "LV-shcontrol" is a non-target shRNA control. The vector "pLKO.1-puro Non-Target shRNA Control Plasmid DNA" (purchased from Sigma, SHC016) contains an shRNA insert that does not target any known genes from any species. The shRNA sequences can be found in Table S9. Lentiviral vectors encoding human KLF7, TLR4, PTK2, and HMGB1 genes were constructed in pLV-puro or pLV-neo (Addgene) and designated as LV-KLF7, LV-TLR4, LV-PTK2 and LV-HMGB1. An empty vector was used as the negative control and was designated as LV-control. The lentivirus and cell infection were produced according to the lentiviral vector protocol recommended by Addgene. Briefly, the lentiviral plasmid and packaging plasmids pMD2.G and psPAX2 (Addgene plasmid #12259 and #12260) were transfected into HEK-293T cells with transfection reagent (Lipofectamine®3000, Thermo Fisher Scientific) and OPTI-MEM media (Invitrogen, Waltham, MA, USA). The lentiviruses were harvested twice on days

4 and 5. Viruses were filtered with a 0.45μm filter and stored at -80 °C. Lentiviral infection of target cells were performed in cell culture media with 5 μg/ mL polybrene (Sigma H9268). Seventy-two hours after infection, cells were selected for 2 weeks using 2.5 μg/mL puromycin (OriGene). Selected pools of cells were used for the following experiments.

Transient transfection

The cells were plated at a density of 1×10^5 cells/well in a 24-well plate. After 12-24 hours, the cells were co-transfected with 0.6 μg of expression vector plasmids, 0.18μg of promoter reporter plasmids, and 0.02 μg of pRL-TK plasmids using Lipofectamine 2000 (Invitrogen, USA) according to the manufacturer's instructions. After 6h of transfection, the cells were washed and allowed to recover overnight in fresh medium supplemented with 1% FBS for 48 h. Serum-starved cells were used for the assay.

Luciferase reporter assay

Luciferase activity was detected using the Dual Luciferase Assay (Promega, USA) according to the manufacturer's instructions. The transfected cells were lysed in culture dishes containing a lysis buffer and the resulting lysates were centrifuged at maximum speed for 1 min in a microcentrifuge. Relative luciferase activity was determined using a ModulusTM TD20/20 Luminometer (Turner Biosystems, USA), and the transfection efficiencies were normalized according to the Renilla activity.

Western blot analysis

Proteins from lysed cells were fractionated by SDS-PAGE and transferred to nitrocellulose membranes. Nonspecific binding sites were blocked with 5% BSA in TBST (120 mM Tris–HCl (pH 7.4), 150 mM NaCl, and 0.05% Tween 20) for 2 hours at room temperature. Blots were incubated with a specific antibody overnight at 4 °C. Western blotting of β-actin on the same membrane was used as a loading control. The membranes were then washed with TBST 3 times and incubated with an HRP-conjugated secondary antibody. Proteins were visualized using an ImmobilonTM Western Chemiluminescent HRP substrate (Millipore, USA).

The primary antibodies used are listed below.

Antibodies	Source
anti-KLF5	Cell signaling technology, #51586
anti-KLF7	Abcam, ab197690
anti-KLF8	Abcam, ab168527
anti-KLF13	Proteintech, 18352-1-AP
anti-TLR4	Proteintech, 66350-1-Ig
anti-PTK2	Cell signaling technology, #3285
anti-RAGE	Abcam, ab216329
anti-NF-κB p65	Cell signaling technology, #8242
anti-p-NF-κB p65 (Ser536)	Cell signaling technology, #3033
anti-Akt	Cell signaling technology, #4691
anti-p-Akt (Ser473)	Cell signaling technology, #4060

anti-JNK	Cell Signaling Technology, #9252
anti-p-JNK (Thr183/Tyr185)	Cell Signaling Technology, #4668
anti-ERK1/2	Cell Signaling Technology, #4695
anti-p-ERK1/2 (Thr202/Tyr204)	Cell Signaling Technology, #4370
anti-p38	Cell Signaling Technology, #8690
anti-p-p38 (Thr180/Tyr182)	Cell signaling technology, #4511
anti-HMGB1	Abcam, ab227526
anti-p-PTK2 (Tyr397)	Cell signaling technology, #3283
anti-β-actin	Proteintech, 60008-1-Ig
anti-GADPH	Cell signaling technology, #5174

Quantitative Real-time PCR (RT-qPCR)

The RNeasy Plus Mini Kit (50) kit (Qiagen, Hilden, Germany) was used to extract total RNA, which was then reverse transcribed with the Advantage RT-for-PCR Kit (Qiagen) in accordance with the manufacturer's protocols. The target sequence was amplified with real-time PCR with the SYBR Green PCR Kit (Qiagen). The cycling parameters used were 95 °C for 15 s, 55-60 °C for 15 s, and 72 °C for 15 s for 45 cycles. Melting curve analyses were performed, and Ct values were determined during the exponential amplification phase of real-time PCR. SDS 1.9.1 software (Applied Biosystems, Massachusetts, USA) was used to evaluate amplification plots. The $2^{-\Delta\Delta Ct}$ method was used to determine relative fold changes in target gene expression in cell lines, which was normalized to expression levels in corresponding control cells (defined

as 1.0). The equation used was $2^{-\Delta\Delta Ct}$ ($\Delta Ct = Ct^{target} - Ct^{ATCB}$; $\Delta\Delta Ct = \Delta Ct^{expressing\ vector} - \Delta Ct^{control\ vector}$). When calculating relative expression levels in surgically extracted HCC samples, relative fold changes in target gene expression were normalized to expression values in normal liver tissues or adjacent nontumor tissues (defined as 1.0) using the following equation: $2^{-\Delta\Delta Ct}$ ($\Delta\Delta Ct = \Delta Ct^{tumor} - \Delta Ct^{nontumor}$). All experiments were performed in duplicate. The primer sequences were listed in Table S8.

Chromatin immunoprecipitation Assay (ChIP)

Cells were immersed in 1% formaldehyde for 10 minutes at 37 °C to stimulate cross-linking. Then, glycine was used to quench the formaldehyde after cross-linking to stop formaldehyde fixation. After washing with PBS, the cells were resuspended in lysis buffer (1 mM PMSF, 1% SDS, 10 mM EDTA and 50 mM Tris (pH 8.1) – total volume 300 μl). Sonication was then performed to produce fragmented DNA. A slurry of protein G-Sepharose and herring sperm DNA (Sigma-Aldrich) was used to clear the supernatant. The recovered supernatant was then subjected to a 2-hour incubation period with specific antibodies or an isotype control IgG in the presence of protein G-Sepharose beads and herring sperm DNA, followed by antibody denaturation with 1% SDS in lysis buffer. Precipitated DNA was extracted from the beads by immersing them in a 1.1 M NaHCO3 solution and 1% SDS solution at 65 °C for 6 hours. Immunoprecipitated DNA was retrieved from the beads by immersion in 1% SDS and a 1.1 M NaHCO3 solution at 65 °C for 6 hours. The DNA was then purified using a PCR Purification Kit (QIAGEN, USA). The primers were shown in Table S8.

For ChIP assays of tissues, cells were first separated from six fresh frozen HCC tissues and three normal liver tissues collected after surgical resection. In detail, surgically extracted tumor tissues were first washed by 1×cold, PBS, 5min, for three times and added to medium supplemented with antibiotic and antifungal agents. Use a clean razor blade to cut a pie of tissue (around $5mm^3$) into small piece (typical $1mm^3$ or smaller). Then, digestion the tissues with DNase I (20 mg/mL; Sigma-Aldrich) and collagenase (1.5 mg/mL; Sigma-Aldrich) and placed on table concentrator, 37°C, for 1h. At the end of the hour, we filtered the dissociated cells through 100-μm-pore filters rinsed with fresh media. The 1×red cell lysis was added to the tissues and incubated for 5 minutes to lysis the red blood cell, followed by another rinse. The dissociated cells were crosslinked using 1% formaldehyde for 10 minutes at 37°C. After cell lysis, the DNA was fragmented by sonication. ChIP grade antibody or IgG (negative control) was used to immunoprecipitated the fragment DNA. Then, qRT-PCR was used to amplify the corresponding binding site on the promoters.

Cell treatment

Established HCC cell lines were seeded in 6-well plates and allowed to attach overnight. Reagents used to treat these cells for 24 hours.

Reagents	Source
IL-1β	PeproTech (10ng/mL)
IL-6	PeproTech (50ng/mL)
IL-8	PeproTech (50ng/mL)

IL-17A	PeproTech (50ng/mL)
TGF-β	PeproTech (5ng/mL)
TNF-α	PeproTech (10ng/mL)
LPS	Sigma-Aldrich (100ng/mL)
HMGB1	R&D Systems (0.25, 0.5, 0.75, 1.0μg/mL)
ERK inhibitor U0126	MedChemExpress (10μM)
JNK inhibitor SP600125	MedChemExpress (20μM)
p38 inhibitor SB203580	MedChemExpress (20μM)
PI3K inhibitor LY294002	MedChemExpress (10μM)
NF-kB inhibitor BAY 11-7082	MedChemExpress (10μM)
TAK-242	MedChemExpress (50μM)
Defactinib	Selleck (10μM)

In vitro migration and invasion assays

Transwell inserts were placed inside 24-well cell culture plates (Corning, USA). The upper chamber was coated with 60μL of Matrigel (Corning, 200 mg/mL) and dried overnight for invasion assays. For the migration and invasion assays, 5×10^4 and 1×10^5 cells, respectively, were plated in the top chamber, and the lower chamber was filled with 600μL of complete medium. After incubation for 24h, the cells on the upper surface of the membranes were removed by swabbing, and the cells on the lower surface were fixed with methanol for 15 min and stained with 0.1% crystal violet for 15min. The average number of cells in five fields per membrane was counted on three inserts.

Colony formation assay

Transfected cells (1000 per well) were cultured in 6-well plates. After 10–14 days of culture, the cells formed stable colonies. The cell colonies were fixed with 70% ethanol and then stained with a crystal violet solution for 10 minutes. Colonies containing more than 50 cells were counted and each group included three replicates.

Construction of tissue microarrays and immunohistochemistry

HCC samples and the corresponding adjacent liver tissues were used to construct a tissue microarray (Shanghai Biochip Co., Ltd. Shanghai, China). IHC was performed on 4-μm-thick, routinely processed paraffin-embedded sections. Briefly, the tissue sections were deparaffinized after baking at 60 °C for an hour. Endogenous peroxidase activity was blocked by 3% (vol/vol) hydrogen peroxide in methanol for 12 min and washes with phosphate-buffered saline (PBS). Then the slides were immersed in 0.01 mol/L citrate buffer solution (pH 6.0) and placed in a microwave oven for 30 min. After washed with PBS, the sections were incubated with bovine serum albumin (BSA) for 30 min, then incubated with the primary antibody diluted in PBS containing 1% (wt/vol) bovine serum albumin in 4 °C for overnight. The tissue microarrays were stained for KLF7 (Sigma-Aldrich, HPA030490, for human; Abcam, ab197690, for mouse), TLR4 (Abcam, ab22048, for human and mice), PTK2 (Cell signaling technology, #3285, for human and mice), HMGB1 (Cell signaling technology, #6893, for human and mice), p-p65 (Abcam, ab86299, for human; ABclonal, AP0475, for mouse). Negative controls

were performed by replacing the primary antibody with preimmune mouse serum. After washed with PBS, the sections were treated with a peroxidase-conjugated second antibody (Santa Cruz) for 30 min at room temperature and then washed with PBS. Reaction product was visualized with diaminobenzidine for 2 min. Images were obtained under a light microscope (Olympus, Japan) equipped with a DP70 digital camera.

Analyses were performed by two independent observers who were blinded to the clinical outcome. The immunostaining intensity was scored on a scale of 0 to 3: 0 (negative), 1 (weak), 2 (medium) or 3 (strong). The percentage of positive cells was evaluated on a scale of 0 to 4: 0 (negative), 1 (1%-25%), 2 (26%-50%), 3 (51%-75%), or 4 (76%-100%). The final immuno-activity scores were calculated by multiplying the above two scores, resulting an overall score which range from 0~12. Each case was ultimately considered "negative" if the final score ranges from 0~3, and "positive" if the final score ranges from 4~12 as described previously [1].

The Human Liver cancer RT^2 profiler PCR array

The Human Liver cancer PCR array was applied to PLC/PRF/5-KLF7 cells and PLC/PRF/5-control cells. RNA extraction, DNase treatment, and RNA cleanup were performed according to the manufacturer's protocol (Qiagen). The cDNA of each group was synthesized using the RT^2 First Strand Kit (Qiagen). Gene expression profiling of PLC/PRF/5-control and PLC/PRF/5-KLF7 cells was conducted using the Human liver cancer RT^2 Profiler PCR Array, which represents 84 genes known to be involved in

liver cancer. The cDNA synthesis reaction was mixed with 2× RT^2 qPCR SYBR Green Mastermix and ddH_2O, and then dispensed to the PCR array 96-well plate (25 μL/well). A 2-step cycling program was performed using the Bio-Rad CFX96. Data normalization was done by correcting all Ct values based on the average Ct values of several housekeeping genes present on array. Each assay was conducted in triplicate.

Klf$^{fl/fl}$ mice and AAV8 administration

The *Klf*$^{fl/fl}$ mice on the C57BL/6J background were created by CRISPR/Cas9-mediated genome engineering. The exon 2 was selected as a conditional knockout region and flanked by LoxP sites and deleted upon Cre-mediated recombination. To induce conditional knockout of *Klf7* in hepatocytes, adeno-associated virus serotype 8 (AAV8) was constructed to express TBG-Cre recombinase in hepatocytes specially. 6-week-old male *Klf*$^{fl/fl}$ mice were given a tail-vein injection of AAV8-TBG-Cre or AAV8-TBG-null at the dose of 5×10^{11} viral gene copies/mouse. To evaluate the Cre recombination efficiency, the expression levels of Klf7 in mice hepatocytes were detected at 1 month and 3 months after AAV8 injection.

Hepatocytes and non-parenchymal cells isolation

Hepatocytes and non-parenchymal cells were isolated after liver perfusion according to previously published protocol [2]. Briefly, the liver was perfused at the speed of 7 mL/min for 4 minutes through the portal vein with perfusion buffer containing collagenase IV. The resulting suspension was centrifuged at 50g and washed 3 times

with sterile Hank's solution, and the hepatocytes were in pellet and the non-parenchymal cells were in supernatant. Cells were then cultured and used for further analysis.

Cytoplasmic protein extraction

Cytoplasmic protein extraction of indicated HCC cells was performed using a Nuclear and Cytoplasmic Protein Extraction Kit (Beyotime, P0027). Briefly, 2×10^6 HCC cells were resuspended in 200 μL of cytoplasmic protein isolation solution A and homogenized on ice for 10 min. Then, 10 μL cytoplasmic protein isolation solution B was added, and the cells were resuspended and homogenized on ice for 1 min. The homogenate was centrifuged at 12000g for 5 min at 4 °C. The resulting supernatant was the cytoplasmic protein fraction.

Bioinformatics analysis

Gene expression or gene signature expression in liver tumors and liver tumor metastatic tissues (Figure 1F, Figure S9A) was analyzed in the TNMplot database (https://tnmplot.com/analysis/) [2]. The expression correlation between two genes in the TCGA LIHC cohort (Figure S7) was performed in the (Gene Expression Profiling Interactive Analysis 2) GEPIA2 database (http://gepia2.cancer-pku.cn/#index) [3]. The prognostic significances of genes and gene sets on overall survival or disease-free survival of HCC patients(Figure S7, Figure S9B) were explored in the Kaplan-Meier Plotter (KM plotter) databases (https://kmplot.com/analysis/) [4].

Figure S1

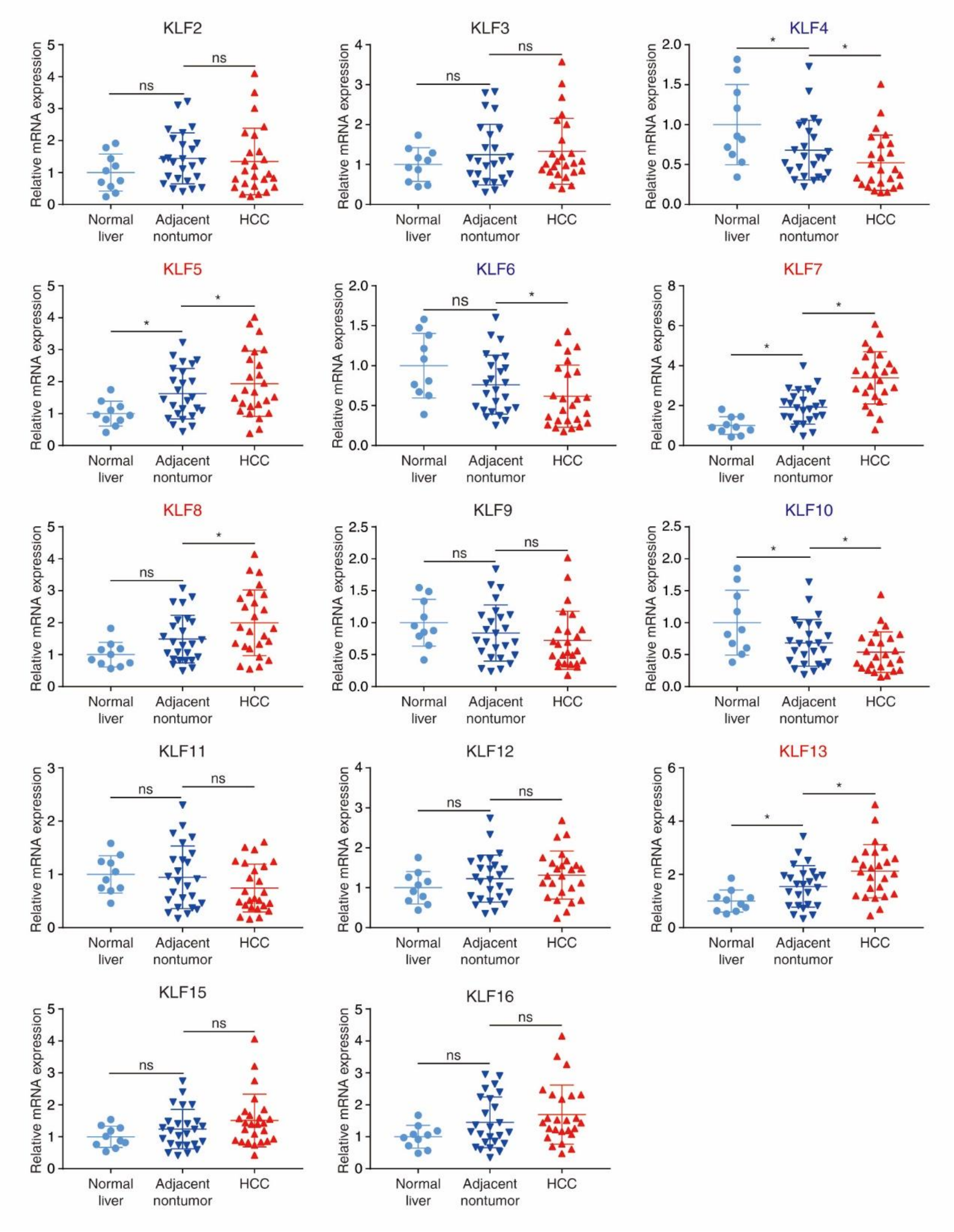

Figure S1

The mRNA levels of *KLF* family genes in normal livers (n = 10) and 25 pairs of adjacent nontumor and HCC tissues were detected by RT-PCR. *KLF7* is the most upregulated *KLF* gene in HCC tissues. *P < 0.05.

Figure S2

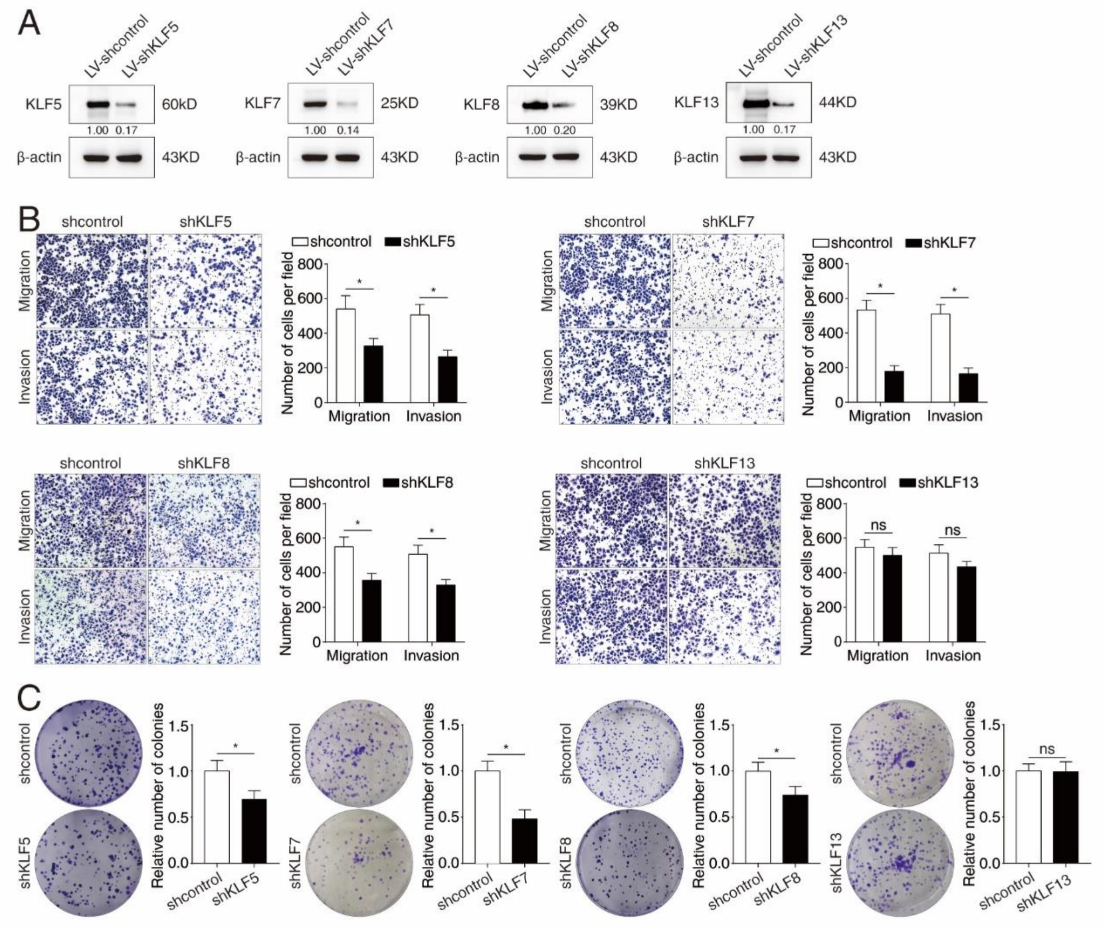

Figure S2

(A) The protein levels of KLF5, KLF7, KLF8, and KLF13 were detected by western blot in MHCC97H cells transfected with shRNA.

(B) Transwell assays were performed to analyze the migration and invasion of MHCC97H cells after the knockdown of KLF5, KLF7, KLF8, and KLF13.

(C) The proliferation abilities of the indicated MHCC97H cells were measured by clone formation assays.

*P < 0.05.

Figure S3

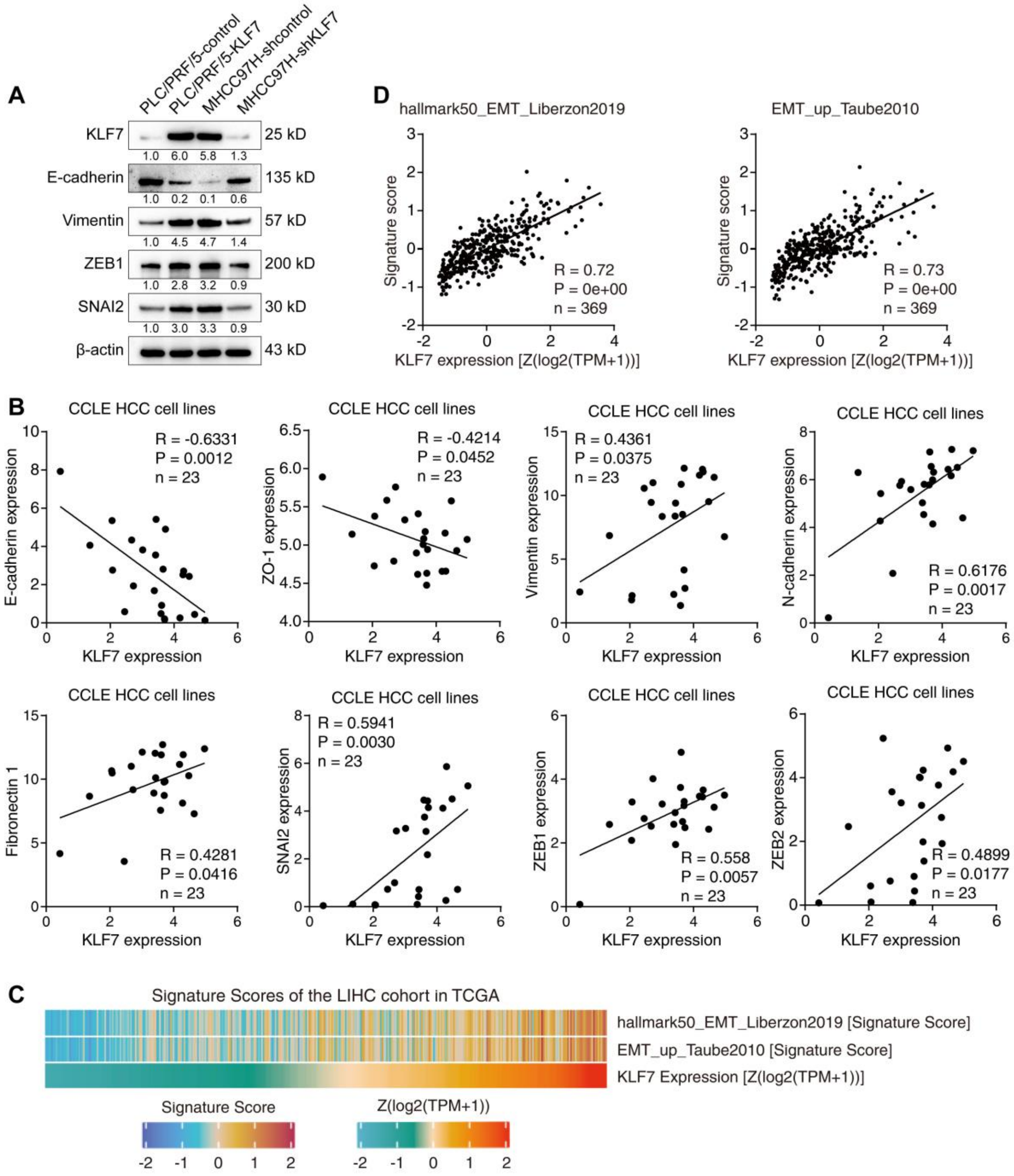

Figure S3

(A) Western blot analysis of epithelial markers (E-cadherin), mesenchymal markers (vimentin) and typical EMT-activating transcription factors (ZEB1, SNAI2) in the indicated HCC cells.

(B) Correlation analysis of *KLF7* expression and the expression of several EMT-related molecules in human HCC cell lines based on CCLE data.

(C) The heatmap displayed the scores of EMT gene signatures and *KLF7* expression in the TCGA LIHC samples.

(D) Correlation analysis between *KLF7* expression and the scores of EMT gene signature in the TCGA LIHC samples.

Figure S4

TLR4 Promoter (-1918 ~ +473)

>NC_000009.12:117702485-117704875 Homo sapiens chromosome 9, GRCh38.p14 Primary Assembly

-1918 AGAAACTTACATTGCACTTGCTACTTTCCAGACACTGTCCTAAAAGCTTTACAAATGCCAGTTCATTTAATCCCAATACAATACTTTGAGA

-1827 TACATATTATCATCTTCATTCTATCCACATTTTCAATCCTCATCATAGCTCTCATTTATGGAATGTAATGATGATGCTCTAGACTAGACGTTTT

-1733 ACGTAAGTTAGCTTAATTCAGTAATTCAAAACACATGCGATTATCTTCGTTTTAAAGACCAGAAAACTAAAGGTTGGTAGGTTTGTATAAT

-1642 TTGACTACCATTGCGTATCTTTATTTTAATACATTTTATAAATGCAAGCTTCTGCTATGATTAAAAGTGATTACCACATTTTACAGACCAGAA

-1549 AGTAATAATAAGTGTTGGTGAAGATGTGAAAAAATGAGAACTCCTGTACACCATTTGTGGGAATGTAAAATGGTACAGATGCTGTGGAGA

-1459 ATCATATGGTGGGTGCTCAAAAAATTAAAAATAGATTTACCACATGATCCAGCAATCTCACTTCTGAGTACGTATCCAAAAGAATTGAAAA

-1368 CAGAGACTTTAAGAGATATTTGTACAACCATGTTTATGGCAGCATTATTCACAATAGCTAACGTGTGGCAACAATGCAAGTGTCCATGAAC

-1277 AGACAAATGGATAAGCAAAATGTGGTCTATACATACAATGGAATATTGTTCAGCTTTAAAAAGGAAGGAGGCTTTGATCTATACTACACAG

-1186 AAAAGAACCTTGAGGACATTATGCAAAGTGAAATAAGCCAGTGACAAAAAGATACATACTGTATGATTCCACTTCTAAGAGCTGCCTAGA

-1096 GTAGTCAAGATTATAGAGACAAAAGTAGTGCATAGATTCAAGGGCCTAGGGAAAGGGGAAATGGGGAGTTATTTATTAATGAATAGTGGT

-1006 GATGATTGTACAAAAATATGAACATAATTAATGCCACTAAATTGTACACATACAAATGGTCAAGATAATAAATTTTATGTTATGTCATGTTAT

-913 GTTATGTGATTTTACCATAATACAGAAAATGAAAAAAGAAAAGAAAGAAAGTAAAGCTTAGCGGTTTACATGACTTGACCAATGCCTCAA

-823 AGCCATGAGTCACCCAGCTGAGATCTGAACTTCAGTATATTCCATTCTGAAATCCCAGACTTTTCCCAATCTTCTTGTACTTTTCAAACTG

-732 TGTTTCAGTTGAGGTTTATTTTCAGTTTTGTATGTGAGTTTCTTCACAAGAAGGGGCGGGCCAAATTGTGTCCTGCAAAAACCTACATATC
KLF7 binding site 3

-641 GAAGTCCTAACCCCTCTACCTCAGACTATGACTGTATATGGAGAGAGAGCCTTGAAAGAGGTATGTAAGGTAGAATGAGGTCATTATGGTG

-550 GGCCCTAATCCAACATAACTGGTGTCCTTATAAGAAGGGGAGATTAGAATTCAGACACACTTGCTGACACCTTGAGTTCAGACTGGAAGC

-460 CTCTAGAATTGTGAGAAAATGAATGTCTGTTGTTTAAGCCACCCAGTCTGTGGTATTTCCTTATGGCAGCCCCAGCAAACTAATACAAATAG

-368 TGTTTCCACAGCTGAAACAAAATTGGAAAATCACCGTCATCCTAGAGAGTTACAAGGGCTATTTTAATAGAACCTGATTGTTTTCCTAAATT

-276 CACCAAGCCCAGGCAGAGGTCAGATGACTAATTGGGATAAAAGCCAACTAGCTTCCTCTTGCTGTTTCTTTAGCCACTGGTCTGCAGGCG
KLF7 binding site 2

-186 TTTTCTTCTTCTAACTTCCTCTCCTGTGACAAAAGAGATAACTATTAGAGAAACAAAAGTCCAGAATGCTAAGGTTGCCGCTTTCACTTCC

-95 TCTCACCCTTTAGCCCAGAACTGCTTTGAATACACCAATTGCTGTGGGGCGGCTCGAGGAAGAGAAGACACCAGTGCCTCAGAAACTGCT
KLF7 binding site 1

-5 CGGTCAGACGGTGATAGCGAGCCACGCATTCACAGGGCCACTGCTGCTCACAGAAGCAGTGAGGATGATGCCAGGATGATGTCTGCCTCG
Transcription starting site (nucleotide+1)

+86 CGCCTGGCTGGGACTCTGATCCCAGCCATGGCCTTCCTCTCCTGCGTGAGACCAGAAAGCTGGGAGCCCTGCGTGGAGGTATGTGGCTGG

+176 AGTCAGCTCCTCTGAACTTTCCCTCACTTCTGCCCAGAACTTCTCACTGTGTGCCCTGGTTTGTTTATTTTTGCAAAAAAAAAAAAGAGTT

+267 AAATTACCTTAAAGACTCAAGAAGCCACAGAGATCAAATAATTCATTGTTACAGGGCACTAGAGGCAGCCATTGGGGGTTTGTTCCATTT

+357 GGAAATTTTGAGTGCTAACAGGGGCATGAGATAACATAGATCTGCTTAAGGTCCCTGCTCTGCTACCTTGTGGCTCTGTGAAGAAATTATC

+448 AAACCTGTCTGAGACTAGTTTTCGCA

Figure S4

KLF7 binding motifs within the *TLR4* promoter. The KLF7 binding sites are marked in yellow and the transcription start site is marked in red.

Figure S5

PTK2 Promoter (-1540 ~ +291)

>NC_000008.11:c141003619-141001789 Homo sapiens chromosome 8, GRCh38.p14 Primary Assembly

-1540 CTGCCCTTCAAGAACGTCAATTTCAAATGTATCGTGGCAGAGTCCAGGAAACAATATTGTGTAACACACTACCCTAA

-1463 AACCTAAAGGCTTAAAACCACATGTATTTTATTGGCTCACAATTCTGTGGTTTGGCAATTTGGGTTGGATTCAGCTGG

-1385 GCAGTTCTTCCATCAGTCTCTCCAGGTCTTATGTATGCTTCACAGGGGCTGGGTGGTCTAGTACAGCTCACTCACATG
KLF7 binding site 3

-1307 TCTGGAGGTGGCACCTTGTTGGCCAGGGTGCCTCAGTTCTCTTCCACACGGCCTCCTCGTGAGGCTAGCTTGGGCTT

-1230 AAGGGGGGAAAATGAAAGCAGCATGGCCTCTTGAGGCCTAGGCTTGGAATTTGTACAATGTCACTTGTGACACATC

-1154 CCATTGGTCAAAGCAAGTTACAAGATCCCATCCACATTCAAGGGGTGAATAAATTCCACCTCTTCTTGGGAAAAGTG

-1077 TCAAAGAATTGATGGCCATTTTTAGTCTACTATGCTAAGTGCAGCCCCTTCCCCCAAAGTTATCCTTAATGCAGTCCCT

-998 TTGTGCCTCCACTCTCCTCTTTTAATTCCCATGCTTTTCTTATCCTTTTAATAATAAGCTCTCACTTCACATCACCCAAA

-918 GCTTCGTTCATTCACTTGACAAATACTTACTGTGTGCCAGGACCTATTAGTGAACAAAACAAAGCCCGTGCTCTAATG

-840 AATCTAAGATTCTAGTGGAGAGAGCGGACAATCAAGAAATAAACAAGAACACAGTGTTGGGCTATGGTGAACAATA

-764 GGTGCTTTGGAGAACAACAGAGCAGACTAAGTGCGGACTGTGTGTGAGGATAGCAGGGCAGTGGGTGGTTCCTATT
KLF7 binding site 2

-688 ATATGAAGTGGTCGGCTGTATCAATGAGGTGATATATAAGCAGGGTATTGAACGAAGTGAGGAGCAAGAAGTTCCAG

-611 GCAGAGCAAATAGCACGCGCAAAGGCCTGAGGCTGGCATGGAGAGGCAATTCCTTACAATTTCATATGTAGTAAAAA

-534 GTAACAAAACCCAGAAAAACACCAACCTTAATACATCTCATTAAAGAACAATACAACCCAAACATTTAAATATGGTA

-457 AAATCTTGATAAGCAAAAGCTTTTTATTTTACTTCATTTTTACTTTTATAAAAGCTCCTAATGCAATGACGTGTGTCAC

-378 CTCTCTGGGCTTCAGGGTGTTCACCTTTCAAAAGGCCTCGGTGCCTGTCTGTCCTGCTTCCTGGCTTGTGGTTAGCAT

-300 CAAAATGACACCATAAAAATGAAAGTTGCAGCCTTGAGGGTTCTTCTGGTTTCATTTCCGACCACAAGGTTGCCAGA

-223 GTGACTTTTTTTTTTTTTTAAATAACACAAATCAGTTATCACTTCCTGCTTAAAGCCCTCCAAAGGTTCCCTGTTGCCT

-144 AAAGAATAAACTCCAGGCACCTAAGCAAGCCCCACGCAGCCGTCTGCATTTTTCAATCCCTCCAACCTCGCCTTTTG

-67 CTATTTCCCCCACCCCACCTACGGCACGTTCCGGTCATAACCAACTAGCTCCTCGCTGAAACTGGCTCACACCGCGG
KLF7 binding site 1 Transcription starting site (nucleotide+1)

+11 AAGCCCGGGTCGGTGTCGGGGCGAGCCTTCCCTCCTTTTCCTGGAGCCCGTCTCAGGTCTGTAGCCCTCGGGAGGGA

+88 TTGCAGGGCTCGTTCCCTGCTGGCGTTCATTTAGGTAACCTGTCTACCTCCAGTGGACTTCTAAGTCCCTTTCATGATT

+167 TTTCATCTTCCAAAATCAAATAATGGCCCAGCCCAACTTAGCTATAATGCCATGAAAAGAATCTCTGAGCCTCAGTTTC

+246 CTTGTCTGCAAAAAGGGCCCAGGAACCGCTCGTTAGGGTCGGTGTG

Figure S5

KLF7 binding motifs within the *PTK2* promoter. The KLF7 binding sites are marked in yellow and the transcription start site is marked in red.

Figure S6

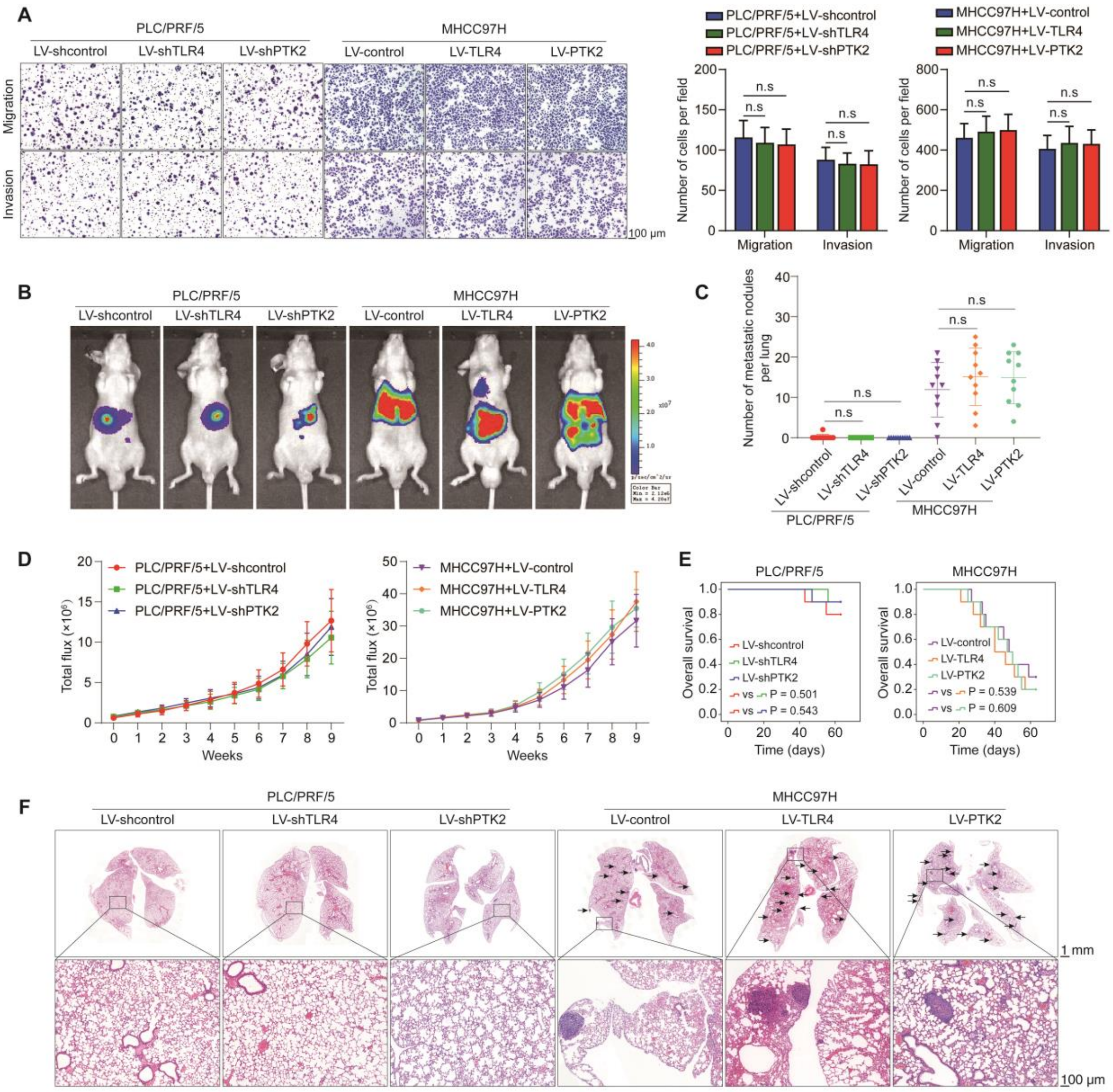

Figure S6. The impact of knockdown or overexpression of TLR4 or PTK2 on lung metastasis was less significant in the KLF7 non-manipulated state than in the KLF7 manipulated state.

(A) TLR4 or PTK2 expression was downregulated in PLC/PRF/5 cells and upregulated in MHCC97H cells through lentiviral transfection. The migratory and invasive

capacities of the indicated HCC cell lines were evaluated by transwell assays.

(B-F) Representative bioluminescent images (B), number of lung-colonizing nodules (C), bioluminescent signals of liver tumors (D), OS (E), and representative HE staining of lung tissues (F) in HCC orthotopic xenograft models.

Figure S7

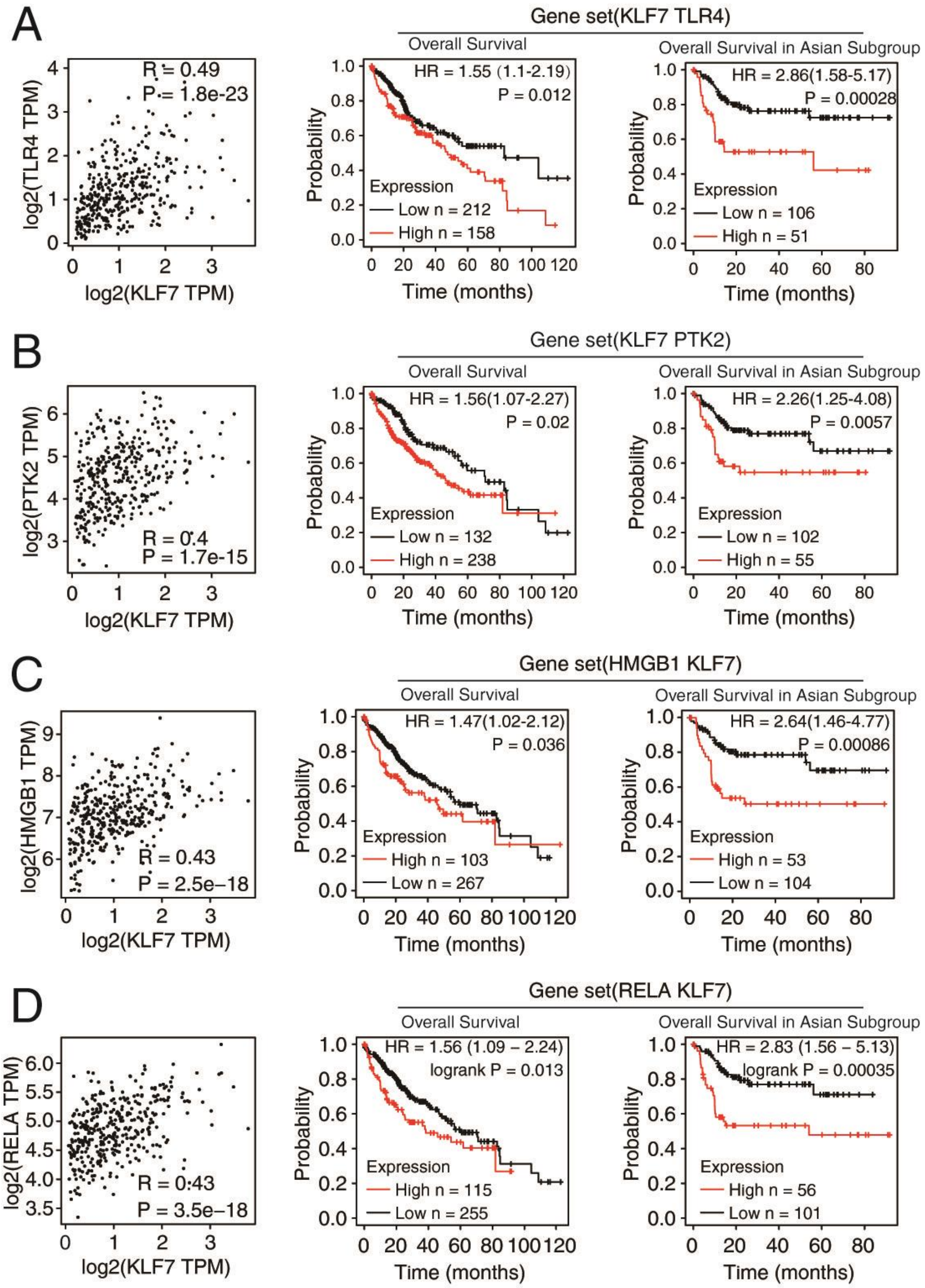

Figure S7

(A) Correlation analysis of *KLF7* expression and *TLR4* expression in the TCGA LIHC samples was performed in the GEPIA2 database. Survival analysis of the mean

expression levels of the *KLF7*/*TLR4* gene set on the overall survival of HCC patients from the TCGA LIHC cohort was performed in the KM-plotter database.

(B) Correlation analysis of *KLF7* expression and *PTK2* expression in the TCGA LIHC samples, and survival analysis of the mean expression levels of the *KLF7*/*PTK2* gene set on the overall survival of HCC patients from the TCGA LIHC cohort.

(C) Correlation analysis of *KLF7* expression and *HMGB1* expression in the TCGA LIHC samples, and survival analysis of the mean expression levels of the *HMGB1*/*KLF7* gene set on the overall survival of HCC patients from the TCGA LIHC cohort.

(D) Correlation analysis of *KLF7* expression and *RELA* expression in the TCGA LIHC samples, and survival analysis of the mean expression levels of the *RELA*/*KLF7* gene set on the overall survival of HCC patients from the TCGA LIHC cohort.

Figure S8

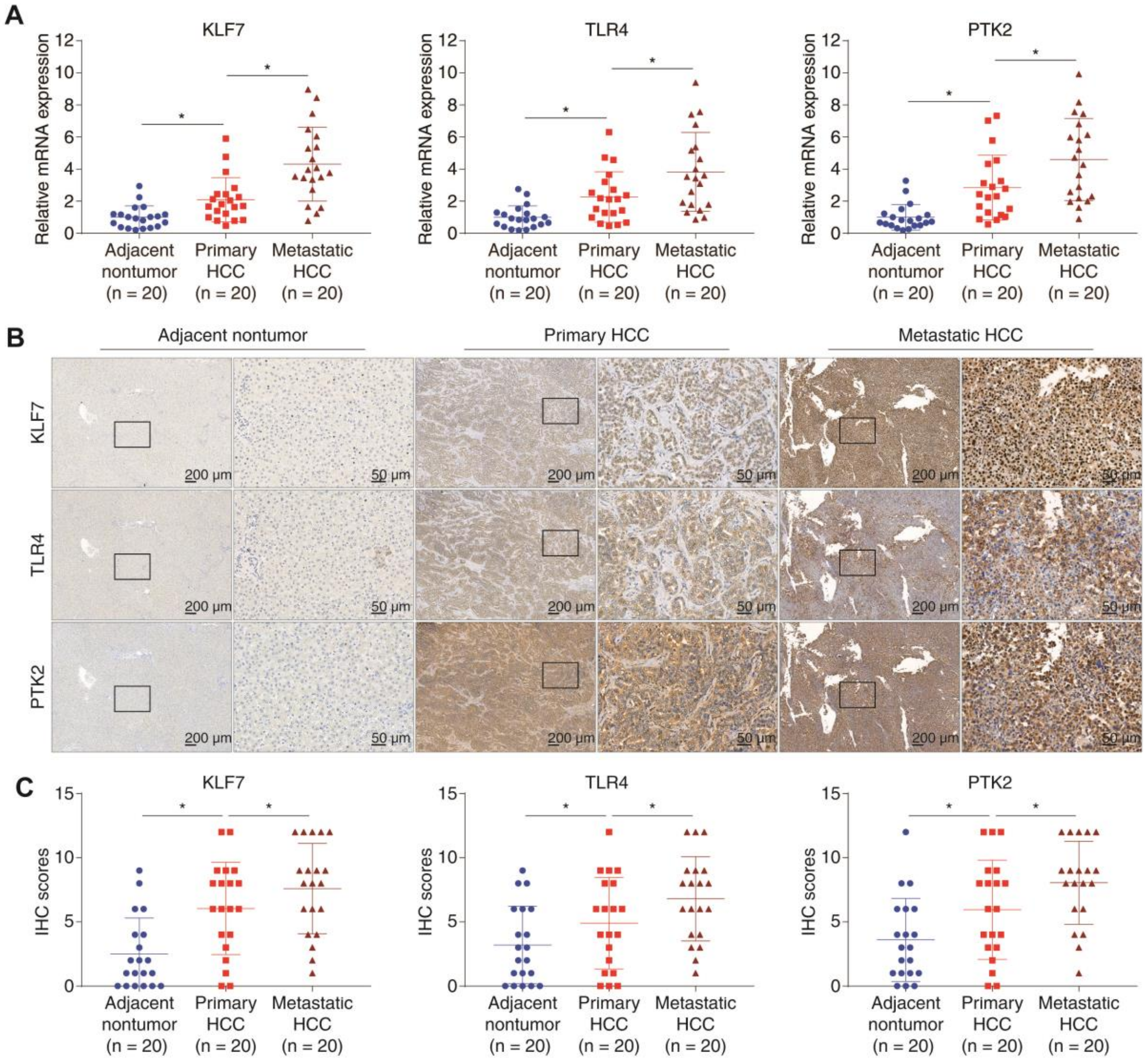

Figure S8

(G) The mRNA levels of *KLF7*, *TLR4*, and *PTK2* in 20 pairs of para-cancer nontumor specimens, primary HCC specimens and metastatic HCC specimens were measured by real-time PCR.

(H-I) Representative IHC staining (H) and IHC scores (I) of KLF7, TLR4 and PTK2 in paired adjacent nonneoplastic tissues, primary HCC tissues and metastatic HCC tissues.

Figure S9

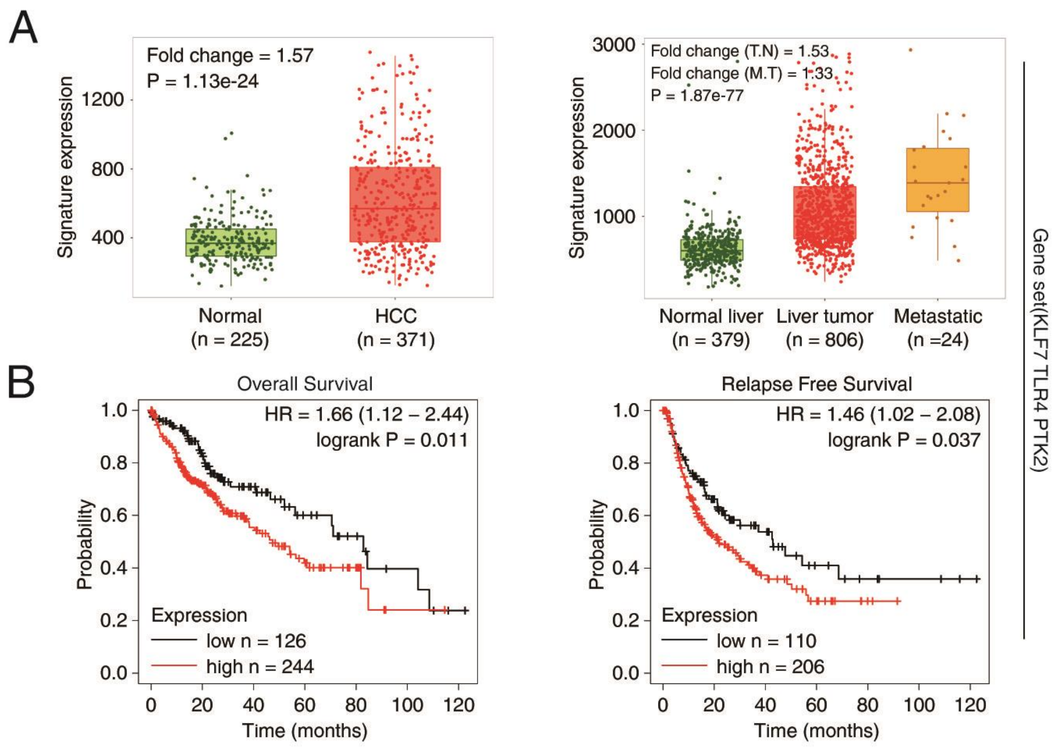

Figure S9

(A) The mean expression of the gene set (KLF7, TLR4, and PTK2) in adjacent normal liver tissues, liver cancer tissues, and liver cancer metastatic tissues. Data from the TNMplot database.

(B) The correlations between the mean expression levels of the gene set (KLF7, TLR4, and PTK2) and overall survival or relapse-free survival in the TCGA LIHC patients were evaluated in the KM-plotter database.

Figure S10

KLF7 Promoter (-1986 ~ +336)

>NC_000002.12:c207175837-207173516 Homo sapiens chromosome 2, GRCh38.p14 Primary Assembly

-1986 ACATGTAGAAAGGACAAAACAAAAGAGGATTGCTTTAACATGAATATTCCTCAAAGATTGAAAATTGATATCACAGAAAAGGAAAGGAT

-1897 TGGGGCTTATATGTGGCACAACGAGAGCTGGGTGGAATTTCCCATTTGGACAAATTGGGTAAGAGGAGTAAGCCTCTCTGAGTTATGCTA
NF-κB binding site 4

-1807 CCTTTTGGTCTTCATCAAATGAGATTGCACTGGCTTCCCAAATAATGACCAAAGGGAGGCAGAGCAGCTAAGGGCATACCTTCCACGATG

-1717 CCCAGTTTGGGAGAAGCCAAAATGAAATGACACAGTATAAAGCATGACCACATTTGCTTCAAACAATCTTTGAGCATTGCAAAGCGGGA
AP-1 binding site 3

-1628 GGAGAAGAGAGTTAGGGGAATTGTGAAGCATTCTGAGAGGGGCATTGAAGCATTATTTCTCCCATTTAGATGGCACAAATGGAGGAATTT

-1538 TTTTTTTTTTTTTTTTTTTTTTTTTTTTGAGGCGGAGTCTCGCTCTGTCGCCCAGGCTGGAGTACACTGGTGCGATCTCGGCTCACTGCAA

-1447 GCTCCGCCTCCCGGGTTCATGCCATTCTCCTGCCTCAGCCTCCGGAGTAGCTGGGACTACAGGCGCCCGCCACCACGCCCGGCTACTTTT

-1357 TTTGCATTTTTTAGTAGAGACGGGGTTTCACCGTGTCAGCCAGGATGGTCTCGATCTCCTGATCTCGTGATCTGCCTGCCTCGGCCTCCCA
NF-κB binding site 3

-1266 AAGTGCTGGGACTACAGGCGTGAGCCACCGCGGCCGGCCCAAATGGAGGATTTTAAAGCATGTGATAACTTACTCCTGAGAAGAGCAG

-1178 GGACCAGCATTAAAAACTATGACTCAGCTGAGTTTTCAAAGCACTCAAGTAAGCATCCTTCTGGTAAAGGAAATTTAGTCAGATTCAAAT
AP-1 binding site 2 **STAT3 binding site 2**

-1088 GAGAGACTAGTCAGAAAAAAAGCAAAACAAAACAAAACCTGCTTCTGACATTATTAATTTTCCAAACTATCTAGATCTATCATATACATAA

-997 AAACTCATCATTGAGCACTTGTGGAATAGGTATCTCTCACTTAAAGGAATGGTCATTGTCAGCAAAGTTTGCAGTACAGAGACTTTCCAT
NF-κB binding site 2

-907 CAAATCAATCTTATTCTCCCATTGATTTCCATTACAAAATCCAAGATGCATTCTGCTGTTAAATCCCATTAACTGCATACCAAGCCCTTTGG

-815 AAATGGCACTTTTAAAAGAAAAATGATAATATTCTGATAAAACATTGAGAGTGATATTTAACATATTAATAATTACACTGTTGTATTCAACAT

-722 GTCACAACAAAAAATGCTTAGGTTGAAACACAACACTCTTACAAATTGTGCCTCACATGCCACAAAAGCTAAGAATCTCTAAAACATTA

-633 GCAACTTGATTTCTCACTGGCTTCACAACTATCTGGGCATTTGCGTCATTGCACAGCAAAATTAATTCTTTTTACTATTTTTTAAATTTTTTT

-540 CTCATTTCCTACTGGCTTCTTTTCCAAAATCTTTGTCCAGAGACCCCTGAATAGCTTGTGATGCTATTCTTAGATGGGGAAAATCATCCAAT
STAT3 binding site 1

-448 CAGGTTCAATCATAGCAGCTTATCCTAGTGCTTGCTGGGATCTCCCTACCATTGGAGGAACTGGGCATCTCCTGAAAAGCAGTGTTGCAA
NF-κB binding site 1

-358 AGGGAAGCAGTAGATCTGATGAAGGTTGGACTAGACACTTCAAATGCCTTTTCTAACTTCTGAGACACAGTACTACTCTCTCAGAAATTG

-268 ATATTGGTAATATGTCTGTGTTTACCATTTTATTTTCAACCTAAGTCCTTTGCATAATATATGTTAAATGACTGGTTTACATAATATATGTTGAA

-173 TGACTGATTGAATTCAAATGATGATTTTGTTCTTCATAGAGGATACTGACACTTTAGGTGCTGACAGCTCAAGCTAGAAAAGTGACTACTA
AP-1 binding site 1

-82 ATATTTATCTCCACAGGAATCCTGTAACACAGGTCATAATGACATTCAAAGTCCAACATTCTAAGGTTGGACAGAGCATTCTTGACAGTGA
Transcription starting site (nucleotide+1)

+10 ACTTTTGCTGGACTTGTGAATTCCTTACCTTTATCATGATGGTAGGTGAATTTTTGTTAAGCAATAGATCACCTGAGTTAGAAGGAAGCAG

+101 CAAATTGTCTCCTGGGTCACACGCCCATAACTCACAATCTACCAGGTCCCTCTTTTATCCATTTTGTATGACCCCTTGCAATGTAAGATGTA

+193 ACATTCAGGGTTGGCTTTGGCATTTTATTTAAAAATAAATGTTTTGTTAAACTGTAATATGCCCACAGAATTCTAAAAGCTAGTGGCAAGAT

+285 TGGGATGTCTTCTGAAGAAGATAATAAGAGTTCTTGGTTAATTCACCGAGAC

Figure S10

The sequence of the *KLF7* promoter. The NF-κB binding sites are highlighted in yellow, the AP-1 binding sites are highlighted in blue, the STAT3 binding sites are highlighted in green, and the transcription start site is marked in red.

Figure S11

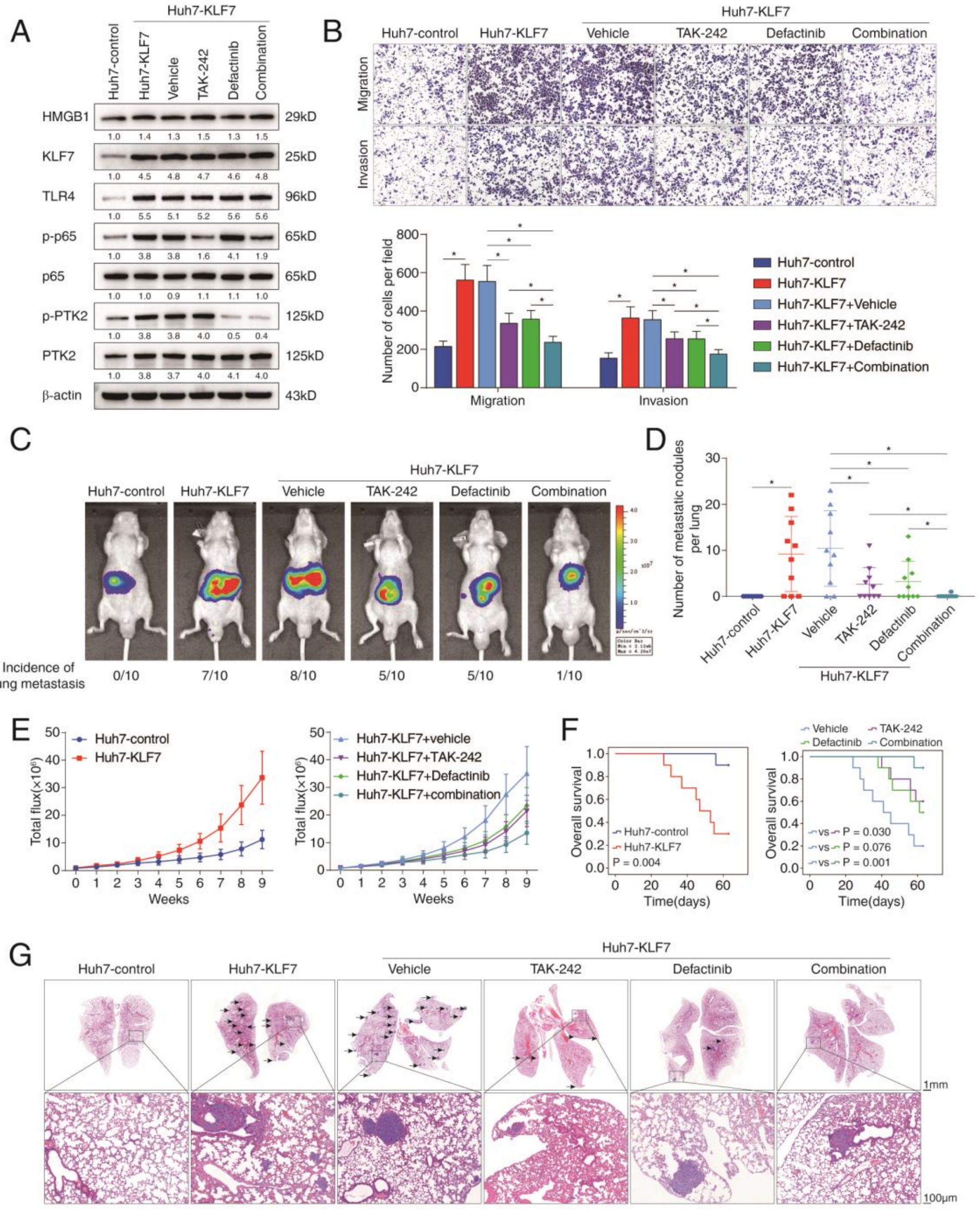

Figure S11. Combined administration of TLR4 and PTK2 inhibitors suppresses KLF7-mediated HCC metastasis.

(A) Lentivirus infection was used to upregulate KLF7 in Huh7 cells, Huh7-KLF7 cells were then incubated with vehicle, TAK-242, defactinib, or a combination of both. The protein levels of HMGB1, KLF7, TLR4, p-p65, p65, p-PTK2, and PTK2 were then

detected.

(B) Transwell analysis of the mobility of Huh7 cells, Huh7-KLF7 cells, and Huh7-KLF7 cells treated with TAK-242 alone, defactinib alone, or a combination of both.

(C-G) Combined blockade of TLR4 and PTK2 alleviated KLF7-mediated HCC metastasis. (C) Representative bioluminescent pictures of liver tumors in nude mice and the rates of lung metastasis. (D) The amount of pulmonary metastatic nodules.

(E) Intensity of bioluminescent signals of liver tumors. (F) Overall survival of mice. (G) Representative pictures of H&E staining of lung tissues.

*$P < 0.05$.

Table S1. Correlation between KLF7 expression and clinicopathological characteristics of HCCs in two independent cohorts of human HCC tissues

Clinicopathological variables		Cohort I			Cohort II		
		Tumor KLF7 expression			Tumor KLF7 expression		
		Negative (n=93)	Positive (n=84)	*P* Value	Negative (n=66)	Positive (n=52)	*P* Value
Age		52.15(12.31)	50.89(11.90)	0.492	54.91(10.77)	53.08(9.94)	0.345
Sex	female	19	16	0.852	17	9	0.371
	male	74	68		49	43	
Serum AFP	≤20ng/mL	25	18	0.483	18	13	0.835
	>20ng/mL	68	66		48	39	
Virus infection	HBV	67	61	0.890	47	35	0.674
	HCV	7	7		8	5	
	HBV+HCV	6	7		2	4	
	none	13	9		9	8	
Cirrhosis	absent	25	17	0.377	22	19	0.846
	present	68	67		44	33	
Child-pugh score	Class A	77	60	0.075	49	39	1.000
	Class B	16	24		17	13	
Tumor number	single	67	38	<0.001	47	27	0.036
	multiple	26	46		19	25	
Maximal tumor size	≤5cm	56	39	0.072	42	23	0.042
	>5cm	37	45		24	29	
Tumor encapsulation	absent	31	44	0.015	18	25	0.022
	present	62	40		48	27	
Microvascular invasion	absent	61	34	0.001	45	20	0.002
	present	32	50		21	32	
Tumor differentiation	I-II	74	38	<0.001	54	32	0.021
	III-Ⅳ	19	46		12	20	
TNM stage	I-II	72	47	0.004	53	32	0.038
	III	21	37		13	20	

Table S2. Univariate and multivariate analysis of factors associated with survival and recurrence in two independent cohorts of human HCC

	Time To Recurrence		Overall Survival	
Clinical Variables	HR(95% CI)	*P* value	HR(95% CI)	*P* value
**Cohort I (n = 177)**				
**Univariate analysis**				
Age	1.005(0.991-1.020)	0.476	1.007(0.992-1.022)	0.371
Sex (female versus male)	0.952(0.618-1.465)	0.823	0.956(0.612-1.494)	0.844
Serum AFP (≤20 versus >20 ng/ml)	0.791(0.520-1.203)	0.273	0.750(0.483-1.164)	0.200
HBV infection (no versus yes)	0.832(0.537-1.289)	0.410	0.688(0.426-1.112)	0.127
Cirrhosis (absent versus present)	0.783(0.511-1.198)	0.259	0.814(0.527-1.255)	0.351
Child-pugh score (A versus B)	0.781(0.523-1.167)	0.228	0.740(0.494-1.108)	0.144
Tumor number (single versus multiple)	0.508(0.357-0.722)	< 0.001	0.463(0.324-0.662)	< 0.001
Maximal tumor size (≤5 versus >5 cm)	0.701(0.494-0.993)	0.046	0.663(0.465-0.946)	0.023
Tumor encapsulation (absent versus present)	2.238(1.575-3.181)	< 0.001	2.321(1.621-3.324)	< 0.001
Microvascular invasion (absent versus present)	0.454(0.318-0.648)	< 0.001	0.451(0.314-0.648)	< 0.001
Tumor differentiation (I-II versus III-IV)	0.422(0.297-0.601)	< 0.001	0.418(0.292-0.598)	< 0.001
TNM stage (I-II versus III)	0.340(0.237-0.486)	< 0.001	0.314(0.218-0.454)	< 0.001
KLF7 (negative versus positive)	0.392(0.275-0.559)	< 0.001	0.394(0.275-0.567)	< 0.001
TLR4 (negative versus positive)	0.434(0.305-0.617)	< 0.001	0.423(0.295-0.606)	< 0.001
PTK2 (negative versus positive)	0.369(0.256-0.530)	< 0.001	0.347(0.238-0.504)	< 0.001
Cytoplasmic HMGB1 (negative versus positive)	0.441(0.310-0.627)	< 0.001	0.438(0.306-0.)	< 0.001
**Multivariate analysis (KLF7)**				
Tumor number (single versus multiple)	0.857(0.572-1.284)	0.454	0.742(0.498-1.106)	0.143
Maximal tumor size (≤5 versus >5 cm)	0.864(0.601-1.242)	0.431	0.829(0.571-1.202)	0.321
Tumor encapsulation (absent versus present)	1.496(1.018-2.199)	0.040	1.502(1.014-2.224)	0.042
Microvascular invasion (absent versus present)	0.650(0.439-0.963)	0.032	0.635(0.426-0.949)	0.027
Tumor differentiation (I-II versus III-IV)	0.713(0.481-1.057)	0.092	0.736(0.492-1.100)	0.135
TNM stage (I-II versus III)	0.517(0.339-0.789)	0.002	0.474(0.313-0.718)	< 0.001
KLF7 (negative versus positive)	0.609(0.407-0.913)	0.016	0.654(0.431-0.990)	0.045
**Multivariate analysis (TLR4)**				
Tumor number (single versus multiple)	0.841(0.561-1.262)	0.404	0.738(0.494-1.103)	0.138
Maximal tumor size (≤5 versus >5 cm)	0.854(0.592-1.231)	0.397	0.827(0.569-1.201)	0.318
Tumor encapsulation (absent versus present)	1.402(0.943-2.084)	0.095	1.386(0.922-2.085)	0.116

Microvascular invasion (absent versus present)	0.633(0.429-0.934)	0.021	0.623(0.419-0.927)	0.019
Tumor differentiation (I-II versus III-IV)	0.654(0.444-0.964)	0.032	0.674(0.453-1.002)	0.051
TNM stage (I-II versus III)	0.523(0.338-0.809)	0.004	0.485(0.316-0.744)	0.001
TLR4 (negative versus positive)	0.594(0.405-0.871)	0.008	0.588(0.397-0.871)	0.008
**Multivariate analysis (PTK2)**				
Tumor number (single versus multiple)	1.054(0.682-1.628)	0.814	0.879(0.578-1.336)	0.545
Maximal tumor size (≤5 versus >5 cm)	0.814(0.566-1.171)	0.267	0.771(0.531-1.119)	0.171
Tumor encapsulation (absent versus present)	1.546(1.054-2.268)	0.026	1.565(1.058-2.315)	0.025
Microvascular invasion (absent versus present)	0.655(0.446-0.964)	0.032	0.651(0.439-0.966)	0.033
Tumor differentiation (I-II versus III-IV)	0.629(0.427-0.925)	0.018	0.655(0.441-0.973)	0.036
TNM stage (I-II versus III)	0.543(0.353-0.835)	0.005	0.525(0.343-0.804)	0.003
PTK2 (negative versus positive)	0.461(0.305-0.696)	< 0.001	0.463(0.305-0.703)	< 0.001
**Multivariate analysis (Cytoplasmic HMGB1)**				
Tumor number (single versus multiple)	0.818(0.551-1.213)	0.317	0.723(0.488-1.070)	0.105
Maximal tumor size (≤5 versus >5 cm)	0.832(0.578-1.198)	0.323	0.785(0.542-1.138)	0.201
Tumor encapsulation (absent versus present)	1.423(0.966-2.098)	0.075	1.433(0.964-2.130)	0.076
Microvascular invasion (absent versus present)	0.620(0.422-0.912)	0.015	0.604(0.408-0.893)	0.012
Tumor differentiation (I-II versus III-IV)	0.685(0.465-1.008)	0.055	0.694(0.469-1.029)	0.069
TNM stage (I-II versus III)	0.491(0.320-0.751)	0.001	0.460(0.301-0.702)	< 0.001
Cytoplasmic HMGB1 (negative versus positive)	0.583(0.402-0.845)	0.004	0.576(0.395-0.840)	0.004
**Cohort II (n = 118)**				
**Univariate analysis**				
Age	0.994(0.974-1.015)	0.588	0.994(0.974-1.015)	0.578
Sex (female versus male)	0.879(0.520-1.485)	0.630	0.884(0.523-1.495)	0.646
Serum AFP (≤20 versus >20 ng/ml)	0.730(0.440-1.210)	0.222	0.740(0.446-1.229)	0.245
HBV infection (no versus yes)	0.731(0.432-1.236)	0.242	0.679(0.397-1.162)	0.158
Cirrhosis (absent versus present)	0.746(0.467-1.190)	0.218	0.697(0.434-1.120)	0.136
Child-pugh score (A versus B)	0.817(0.506-1.318)	0.407	0.783(0.485-1.265)	0.317
Tumor number (single versus multiple)	0.372(0.238-0.580)	< 0.001	0.360(0.230-0.563)	< 0.001
Maximal tumor size (≤5 versus >5 cm)	0.575(0.370-0.893)	0.014	0.575(0.369-0.897)	0.015
Tumor encapsulation (absent versus present)	2.192(1.406-3.418)	0.001	2.187(1.400-3.418)	0.001
Microvascular invasion (absent versus present)	0.372(0.238-0.581)	< 0.001	0.365(0.233-0.571)	< 0.001
Tumor differentiation (I-II versus III-IV)	0.231(0.145-0.371)	< 0.001	0.227(0.142-0.364)	< 0.001

TNM stage (I-II versus III)	0.297(0.187-0.473)	< 0.001	0.298(0.187-0.474)	< 0.001
KLF7 (negative versus positive)	0.418(0.269-0.650)	< 0.001	0.423(0.271-0.658)	< 0.001
TLR4 (negative versus positive)	0.425(0.273-0.660)	< 0.001	0.411(0.264-0.641)	< 0.001
PTK2 (negative versus positive)	0.457(0.290-0.720)	0.001	0.438(0.277-0.694)	< 0.001
Cytoplasmic HMGB1 (negative versus positive)	0.349(0.224-0.544)	< 0.001	0.343(0.220-0.537)	< 0.001
**Multivariate analysis (KLF7)**				
Tumor number (single versus multiple)	0.532(0.331-0.853)	0.009	0.526(0.327-0.847)	0.008
Maximal tumor size (≤5 versus >5 cm)	0.768(0.478-1.233)	0.274	0.744(0.461-1.201)	0.226
Tumor encapsulation (absent versus present)	1.102(0.660-1.840)	0.710	1.079(0.644-1.808)	0.772
Microvascular invasion (absent versus present)	0.570(0.350-0.928)	0.024	0.552(0.337-0.905)	0.018
Tumor differentiation (I-II versus III-IV)	0.351(0.205-0.603)	< 0.001	0.350(0.203-0.603)	< 0.001
TNM stage (I-II versus III)	0.394(0.238-0.650)	< 0.001	0.408(0.245-0.678)	0.001
KLF7 (negative versus positive)	0.614(0.387-0.973)	0.038	0.627(0.393-0.998)	0.049
**Multivariate analysis (TLR4)**				
Tumor number (single versus multiple)	0.489(0.305-0.782)	0.003	0.486(0.303-0.779)	0.003
Maximal tumor size (≤5 versus >5 cm)	0.702(0.441-1.116)	0.134	0.669(0.420-1.065)	0.090
Tumor encapsulation (absent versus present)	1.056(0.642-1.739)	0.829	1.027(0.621-1.698)	0.918
Microvascular invasion (absent versus present)	0.571(0.351-0.931)	0.025	0.560(0.342-0.917)	0.021
Tumor differentiation (I-II versus III-IV)	0.388(0.225-0.669)	0.001	0.386(0.224-0.666)	0.001
TNM stage (I-II versus III)	0.417(0.250-0.694)	0.001	0.434(0.259-0.726)	0.002
TLR4 (negative versus positive)	0.681(0.422-1.101)	0.117	0.652(0.402-1.057)	0.083
**Multivariate analysis (PTK2)**				
Tumor number (single versus multiple)	0.500(0.313-0.800)	0.004	0.498(0.311-0.799)	0.004
Maximal tumor size (≤5 versus >5 cm)	0.721(0.455-1.142)	0.163	0.686(0.432-1.089)	0.110
Tumor encapsulation (absent versus present)	1.024(0.614-1.707)	0.929	0.994(0.593-1.666)	0.982
Microvascular invasion (absent versus present)	0.540(0.334-0.872)	0.012	0.525(0.324-0.853)	0.009
Tumor differentiation (I-II versus III-IV)	0.389(0.221-0.682)	0.001	0.390(0.222-0.685)	0.001
TNM stage (I-II versus III)	0.383(0.230-0.638)	< 0.001	0.396(0.237-0.664)	< 0.001
PTK2 (negative versus positive)	0.768(0.461-1.281)	0.312	0.743(0.444-1.242)	0.257
**Multivariate analysis (Cytoplasmic HMGB1)**				
Tumor number (single versus multiple)	0.561(0.350-0.899)	0.016	0.552(0.344-0.886)	0.014
Maximal tumor size (≤5 versus >5 cm)	0.707(0.441-1.134)	0.151	0.675(0.421-1.084)	0.104
Tumor encapsulation (absent versus present)	1.061(0.634-1.777)	0.822	1.051(0.626-1.764)	0.851
Microvascular invasion (absent versus present)	0.583(0.357-0.951)	0.031	0.558(0.340-0.917)	0.021

Tumor differentiation (I-II versus III-IV)	0.466(0.264-0.821)	0.008	0.475(0.269-0.840)	0.011
TNM stage (I-II versus III)	0.307(0.179-0.529)	< 0.001	0.317(0.183-0.549)	< 0.001
Cytoplasmic HMGB1 (negative versus positive)	0.448(0.267-0.751)	0.002	0.446(0.265-0.749)	0.002

Table S3. List of genes differentially expressed in PLC/PRF/5-KLF7 versus PLC/PRF/5-control cells using a human liver cancer PCR array

gene	Fold change	Description
TLR4	7.31	Toll-like receptor 4
PTK2	6.82	PTK2 protein tyrosine kinase 2
TCF4	5.27	Transcription factor 4
ITGB1	4.73	Integrin, beta 1 (fibronectin receptor, beta polypeptide, antigen CD29 includes MDF2, MSK12)
FZD7	4.29	Frizzled family receptor 7
ADAM17	4.07	ADAM metallopeptidase domain 17
RAC1	3.86	Ras-related C3 botulinum toxin substrate 1 (rho family, small GTP binding protein Rac1)
XIAP	3.62	X-linked inhibitor of apoptosis
BCL2	3.37	B-cell CLL/lymphoma 2
CCL5	3.03	Chemokine (C-C motif) ligand 5
MCL1	2.66	Myeloid cell leukemia sequence 1 (BCL2-related)
NRAS	2.41	Neuroblastoma RAS viral (v-ras) oncogene homolog
YAP1	2.24	Yes-associated protein 1
TGFA	2.09	Transforming growth factor, alpha
TNFRSF10B	1.98	Tumor necrosis factor receptor superfamily, member 10b
VEGFA	1.96	Vascular endothelial growth factor A
KDR	1.91	Kinase insert domain receptor (a type III receptor tyrosine kinase)
TGFBR2	1.89	Transforming growth factor, beta receptor II (70/80kDa)
E2F1	1.86	E2F transcription factor 1
RHOA	1.76	Ras homolog gene family, member A
HGF	1.65	Hepatocyte growth factor (hepapoietin A; scatter factor)
CXCR4	1.56	Chemokine (C-X-C motif) receptor 4
TGFB1	1.51	Transforming growth factor, beta 1
IRS1	1.49	Insulin receptor substrate 1
IGF2	1.46	Insulin-like growth factor 2 (somatomedin A)
STAT3	1.45	Signal transducer and activator of transcription 3 (acute-phase response factor)
CCND2	1.42	Cyclin D2
EGF	1.39	Epidermal growth factor
LEF1	1.37	Lymphoid enhancer-binding factor 1
FLT1	1.35	Fms-related tyrosine kinase 1 (vascular endothelial growth factor/vascular permeability factor receptor)
SMAD4	1.33	SMAD family member 4
MET	1.32	Met proto-oncogene (hepatocyte growth factor receptor)
CTNNB1	1.32	Catenin (cadherin-associated protein), beta 1, 88kDa
AKT1	1.29	V-akt murine thymoma viral oncogene homolog 1

IGFBP3	1.28	Insulin-like growth factor binding protein 3
TERT	1.28	Telomerase reverse transcriptase
EGFR	1.27	Epidermal growth factor receptor
PDGFRA	1.25	Platelet-derived growth factor receptor, alpha polypeptide
PTGS2	1.25	Prostaglandin-endoperoxide synthase 2 (prostaglandin G/H synthase and cyclooxygenase)
ANGPT2	1.24	Angiopoietin 2
EP300	1.21	E1A binding protein p300
MYC	1.21	V-myc myelocytomatosis viral oncogene homolog (avian)
SMAD7	1.20	SMAD family member 7
TNFSF10	1.18	Tumor necrosis factor (ligand) superfamily, member 10
IGFBP1	1.17	Insulin-like growth factor binding protein 1
NFKB1	1.15	Nuclear factor of kappa light polypeptide gene enhancer in B-cells 1
MTDH	1.12	Metadherin
BIRC2	1.12	Baculoviral IAP repeat containing 2
CCND1	1.10	Cyclin D1
HRAS	1.08	V-Ha-ras Harvey rat sarcoma viral oncogene homolog
HHIP	1.06	Hedgehog interacting protein
SFRP2	1.05	Secreted frizzled-related protein 2
PYCARD	1.03	PYD and CARD domain containing
GSTP1	1.01	Glutathione S-transferase pi 1
GADD45B	-1.03	Growth arrest and DNA-damage-inducible, beta
CDH13	-1.03	Cadherin 13, H-cadherin (heart)
FHIT	-1.05	Fragile histidine triad gene
WT1	-1.07	Wilms tumor 1
RUNX3	-1.09	Runt-related transcription factor 3
BCL2L1	-1.12	BCL2-like 1
RASSF1	-1.17	Ras association (RalGDS/AF-6) domain family member 1
FAS	-1.18	Fas (TNF receptor superfamily, member 6)
BID	-1.23	BH3 interacting domain death agonist
BIRC5	-1.25	Baculoviral IAP repeat containing 5
FADD	-1.26	Fas (TNFRSF6)-associated via death domain
PIN1	-1.29	Peptidylprolyl cis/trans isomerase, NIMA-interacting 1
DLC1	-1.32	Deleted in liver cancer 1
OPCML	-1.33	Opioid binding protein/cell adhesion molecule-like
CDKN2A	-1.40	Cyclin-dependent kinase inhibitor 2A (melanoma, p16, inhibits CDK4)
MSH2	-1.44	MutS homolog 2, colon cancer, nonpolyposis type 1 (E. coli)
RB1	-1.47	Retinoblastoma 1
PTEN	-1.53	Phosphatase and tensin homolog
SOCS1	-1.64	Suppressor of cytokine signaling 1
TP53	-1.67	Tumor protein p53
DAB2IP	-1.75	DAB2 interacting protein

CDH1	-1.88	Cadherin 1, type 1, E-cadherin (epithelial)
CDKN1A	-1.97	Cyclin-dependent kinase inhibitor 1A (p21, Cip1)
BAX	-2.06	BCL2-associated X protein
SOCS3	-2.10	Suppressor of cytokine signaling 3
CFLAR	-2.19	CASP8 and FADD-like apoptosis regulator
RELN	-2.31	Reelin
MSH3	-2.54	MutS homolog 3 (E. coli)
CASP8	-2.93	Caspase 8, apoptosis-related cysteine peptidase
CDKN1B	-3.27	Cyclin-dependent kinase inhibitor 1B (p27, Kip1)

Table S4. Correlation between TLR4 expression and clinicopathological characteristics of HCCs in two independent cohorts of human HCC tissues

Clinicopathological variables		Cohort I Tumor TLR4 expression Negative (n=98)	Cohort I Tumor TLR4 expression Positive (n=79)	Cohort I *P* Value	Cohort II Tumor TLR4 expression Negative (n=65)	Cohort II Tumor TLR4 expression Positive (n=53)	Cohort II *P* Value
Age		50.92(11.94)	52.43(12.34)	0.438	53.06(9.57)	55.38(11.32)	0.231
Sex	female	19	16	1.000	12	14	0.373
	male	79	63		53	39	
Serum AFP	≤20ng/mL	22	21	0.598	19	12	0.529
	>20ng/mL	76	58		46	41	
Virus infection	HBV	76	52	0.239	45	37	0.957
	HCV	8	6		8	5	
	HBV+HCV	5	8		3	3	
	none	9	13		9	8	
Cirrhosis	absent	22	20	0.723	25	16	0.437
	present	76	59		40	37	
Child-pugh score	Class A	74	63	0.589	49	39	0.835
	Class B	24	16		16	14	
Tumor number	single	66	39	0.021	44	30	0.253
	multiple	32	40		21	23	
Maximal tumor size	≤5cm	56	39	0.363	40	25	0.139
	>5cm	42	40		25	28	
Tumor encapsulation	absent	31	44	0.001	18	25	0.035
	present	67	35		47	28	
Microvascular invasion	absent	61	34	0.015	43	22	0.009
	present	37	45		22	31	
Tumor differentiation	I-II	69	43	0.041	55	31	0.002
	III-Ⅳ	29	36		10	22	
TNM stage	I-II	76	43	0.001	55	30	0.001
	III	22	36		10	23	

Table S5. Correlation between PTK2 expression and clinicopathological characteristics of HCCs in two independent cohorts of human HCC tissues

Clinicopathological variables		Cohort I Tumor PTK2 expression Negative (n=85)	Cohort I Tumor PTK2 expression Positive (n=92)	Cohort I *P* Value	Cohort II Tumor PTK2 expression Negative (n=55)	Cohort II Tumor PTK2 expression Positive (n=63)	Cohort II *P* Value
Age		51.14(11.89)	51.93(12.35)	0.664	54.55(10.05)	53.71(10.78)	0.667
Sex	female	19	16	0.453	15	11	0.266
	male	66	76		40	52	
Serum AFP	≤20ng/mL	21	22	1.000	14	17	1.000
	>20ng/mL	64	70		41	46	
Virus infection	HBV	62	66	0.942	38	44	0.193
	HCV	6	8		9	4	
	HBV+HCV	7	6		3	3	
	none	10	12		5	12	
Cirrhosis	absent	19	23	0.726	19	22	1.000
	present	66	69		36	41	
Child-pugh score	Class A	68	69	0.475	42	46	0.832
	Class B	17	23		13	17	
Tumor number	single	67	38	<0.001	41	33	0.014
	multiple	18	54		14	30	
Maximal tumor size	≤5cm	47	48	0.763	33	32	0.357
	>5cm	38	44		22	31	
Tumor encapsulation	absent	29	46	0.035	13	30	0.008
	present	56	46		42	33	
Microvascular invasion	absent	57	38	0.001	39	26	0.002
	present	28	54		16	37	
Tumor differentiation	I-II	63	49	0.005	50	36	<0.001
	III-Ⅳ	22	43		5	27	
TNM stage	I-II	69	50	<0.001	45	40	0.039
	III	16	42		10	23	

Table S6. Correlation between cytoplasmic HMGB1 expression and clinicopathological characteristics of HCCs in two independent cohorts of human HCC tissues

		Cohort I			Cohort II		
		Tumor cytoplasmic HMGB1 expression			Tumor cytoplasmic HMGB1 expression		
Clinicopathological variables		Negative (n=103)	Positive (n=74)	*P* Value	Negative (n=68)	Positive (n=50)	*P* Value
Age		50.89(12.43)	52.47(11.66)	0.393	54.28(11.13)	53.86(9.46)	0.830
Sex	female	20	15	1.000	14	12	0.661
	male	83	59		54	38	
Serum AFP	≤20ng/mL	23	20	0.483	20	11	0.404
	>20ng/mL	80	54		48	39	
Virus infection	HBV	68	60	0.178	45	37	0.200
	HCV	10	4		10	3	
	HBV+HCV	9	4		5	1	
	none	16	6		8	9	
Cirrhosis	absent	26	16	0.597	25	16	0.696
	present	77	58		43	34	
Child-pugh score	Class A	79	58	0.857	50	38	0.832
	Class B	24	16		18	12	
Tumor number	single	70	35	0.008	50	24	0.007
	multiple	33	39		18	26	
Maximal tumor size	≤5cm	59	36	0.287	39	26	0.580
	>5cm	44	38		29	24	
Tumor encapsulation	absent	36	39	0.021	19	24	0.033
	present	67	35		49	26	
Microvascular invasion	absent	64	31	0.009	44	21	0.016
	present	39	43		24	29	
Tumor differentiation	I-II	72	40	0.040	58	28	0.001
	III-Ⅳ	31	34		10	22	
TNM stage	I-II	79	40	0.002	54	31	0.041
	III	24	34		14	19	

Table S7. Correlation between p-p65 expression and clinicopathological characteristics of HCCs in two independent cohorts of human HCC tissues

		Cohort I			Cohort II		
		Tumor p-p65 expression			Tumor p-p65 expression		
Clinicopathological variables		Negative (n=82)	Positive (n=95)	*P* Value	Negative (n=54)	Positive (n=64)	*P* Value
Age		51.27(12.55)	51.80(11.77)	0.772	55.11(9.47)	53.25(11.15)	0.336
Sex	female	16	19	1.000	13	13	0.661
	male	66	76		41	51	
Serum AFP	≤20ng/mL	21	22	0.728	16	15	0.530
	>20ng/mL	61	73		38	49	
Virus infection	HBV	60	68	0.913	38	44	0.486
	HCV	6	8		8	5	
	HBV+HCV	5	8		2	4	
	none	11	11		6	11	
Cirrhosis	absent	16	26	0.288	20	21	0.700
	present	66	69		34	43	
Child-pugh score	Class A	67	70	0.214	38	50	0.398
	Class B	15	25		16	14	
Tumor number	single	56	49	0.032	41	33	0.008
	multiple	26	46		13	31	
Maximal tumor size	≤5cm	51	44	0.049	36	29	0.026
	>5cm	31	51		18	35	
Tumor encapsulation	absent	25	50	0.004	12	31	0.004
	present	57	45		42	33	
Microvascular invasion	absent	57	38	<0.001	36	29	0.026
	present	25	57		18	35	
Tumor differentiation	I-II	61	51	0.005	46	40	0.007
	III-Ⅳ	21	44		8	24	
TNM stage	I-II	64	55	0.006	46	39	0.004
	III	18	40		8	25	

Table S8. Primer sequences used in the study

Primer name	Primer sequences	Enzyme
Primers for real-time PCR:		
KLF1 sense:	5’-GGTTGCGGCAAGAGCTACA-3’	
KLF1 antisense:	5’-GTCAGAGCGCGAAAAAGCAC-3’	
KLF2 sense:	5’-TTCGGTCTCTTCGACGACG-3’	
KLF2 antisense:	5’-TGCGAACTCTTGGTGTAGGTC-3’	
KLF3 sense:	5’-GCACGGAATACAGATGGAGCC-3’	
KLF3 antisense:	5’-TGTGAGGACGGGAACTTCAGA-3’	
KLF4 sense:	5’-CAGCTTCACCTATCCGATCCG-3’	
KLF4 antisense:	5’-GACTCCCTGCCATAGAGGAGG-3’	
KLF5 sense:	5’-CCTGGTCCAGACAAGATGTGA-3’	
KLF5 antisense:	5’-GAACTGGTCTACGACTGAGGC-3’	
KLF6 sense:	5’-TCTCATCAGCCCGAGCTTTTG-3’	
KLF6 antisense:	5’-GAGCTGTCAGAGGATTCGCT-3’	
KLF7 sense	5’-CTCGGCAGTGGACATCTTG-3’	
KLF7 antisense	5’-CACCAGTTTCAACGTCACCGT-3’	
KLF8 sense	5’-CCCAAGTGGAACCAGTTGACC-3’	
KLF8 antisense	5’-GACGTGGACACCACAAGGG-3’	
KLF9 sense	5’-GCCGCCTACATGGACTTCG-3’	
KLF9 antisense	5’-GGATGGGTCGGTACTTGTTCA-3’	
KLF10 sense	5’-ACTGCCAAACCTCACATTGC-3’	
KLF10 antisense	5’-ACGAATCACACTTGTTGCCTG-3’	
KLF11 sense	5’-GTTGCGGATAAGACCCCTCAC-3’	
KLF11 antisense	5’-TGGAATCTGTTACTTGGGGAGA-3’	
KLF12 sense	5’-CCCCGGAGGACTCGTTATCT-3’	
KLF12 antisense	5’-GGGAGGATGAAACGGCAGTAG-3’	
KLF13 sense	5’-CGGCCTCAGACAAAGGGTC-3’	
KLF13 antisense	5’-TTCCCGTAAACTTTCTCGCAG-3’	
KLF14 sense	5’-TTACAAGTCGTCGCACCTCAA-3’	
KLF14 antisense	5’-TCTGGATGATAGGTTGGGTGG-3’	
KLF15 sense	5’-TTCTCGTCGCCAAAATGCC-3’	
KLF15 antisense	5’-CCTGGGACAATAGGAAGTCCAA-3’	
KLF16 sense	5’-TTCTCGTCGCCAAAATGCC-3’	
KLF16 antisense	5’-CCTGGGACAATAGGAAGTCCAA-3’	
KLF17 sense	5’-GCTGCCCAGGATAACGAGAAC-3’	
KLF17 antisense	5’-ATCTCTGCGCTGTGAGGAAAG-3’	
TLR4 sense:	5’-AGACCTGTCCCTGAACCCTAT-3’	
TLR4 antisense:	5’-CGATGGACTTCTAAACCAGCCA-3’	
PTK2 sense:	5’-GCTTACCTTGACCCCAACTTG-3’	
PTK2 antisense:	5’-ACGTTCCATACCAGTACCCAG-3’	
β-actin sense:	5’-CATGTACGTTGCTATCCAGGC-3’	
β-actin antisense:	5’-CTCCTTAATGTCACGCACGAT-3’	
Primers for *TLR4* promoter construct:		

(-1918/+473) *TLR4* sense:	5'-TATAGAGCTCAGAAACTTACATTGCACT-3'	SacI
(-464/+473) *TLR4* sense:	5'-TATAGAGCTCAAGCCTCTAGAATTGTGA-3'	SacI
(-228/+473) *TLR4* sense:	5'-TATAGAGCTCTAGCTTCCTCTTGCTGTT-3'	SacI
(-40/+473) *TLR4* sense:	5'-TATAGAGCTCGAGGAAGAGAAGACACCA-3'	SacI
Antisense:	5'-ATATGCTAGCTGCGAAAACTAGTCTCAG-3'	NheI
Primers for *TLR4* promoter site-directed mutagenesis:		
KLF7 binding site:		
binding site 3 mutation sense:	5'-TTCACAAGAAGG**tata**GGCCAAATTGTGT-3'	
binding site 3 mutation antisense:	5'-ACACAATTTGGCC**tata**CCTTCTTGTGAA-3'	
binding site 2 mutation sense:	5'-CCTAAATTCACC**gcta**CCAGGCAGAGGTC-3'	
binding site 2 mutation antisense:	5'-GACCTCTGCCTGG**tagc**GGTGAATTTAGG-3'	
binding site 1 mutation sense:	5'-CAATTGCTGTGG**tata**GCTCGAGGAAGAG-3'	
binding site 1 mutation antisense:	5'-CTCTTCCTCGAGC**tata**CCACAGCAATTG-3'	
Primers used for ChIP in the *TLR4* promoter:		
distant region sense:	5'-TGGCTAGTTTGAATAACCTC-3'	
distant region antisense:	5'-CTTATGCACACTAAGCAA-3'	
binding site sense:	5'-AGACTTTTCCCAATCTTC-3'	
binding site antisense:	5'-TACCTCTTTCAAGGCTCT-3'	
Primers for *PTK2* promoter construct:		
(-1540/+291) *PTK2* sense:	5'-TATAGAGCTCCTGCCCTTCAAGAACGTC-3'	SacI
(-1008/+291) *PTK2* sense:	5'-TATAGAGCTCTGCAGTCCCTTTGTGCC-3'	SacI
(-247/+291) *PTK2* sense:	5'-TATAGAGCTCATTTCCGACCACAAGGTT-3'	SacI
(-42/+291) *PTK2* sense:	5'-TATAGAGCTCACGTTCCGGTCATAACCA-3'	SacI
Antisense:	5'-ATATAGATCTCACACCGACCCTAACGAG-3'	BglII
Primers for *PTK2* promoter site-directed mutagenesis:		
KLF7 binding site:		
binding site 3 mutation sense:	5'-ATGCTTCACAGG**tatg**GGGTGGTCTAGTA-3'	
binding site 3 mutation antisense:	5'-TACTAGACCACCC**cata**CCTGTGAAGCAT-3'	
binding site 2 mutation sense:	5'-TGAGGATAGCAG**taag**GTGGGTGGTTCCT-3'	
binding site 2 mutation antisense:	5'-AGGAACCACCCAC**ctta**CTGCTATCCTCA-3'	
binding site 1 mutation sense:	5'-TTGCTATTTCCCC**agta**CCACCTACGGCA-3'	
binding site 1 mutation antisense:	5'-TGCCGTAGGTGG**tact**GGGGAAATAGCAA-3'	
Primers used for ChIP in the *PTK2* promoter:		
distant region sense:	5'-TAGCGTCTACATCACAGGGTT-3'	
distant region antisense:	5'-CCAGAGCACAGAGTCAAGGA-3'	
binding site 3 sense:	5'-GGCAGTTCTTCCATCAGT-3'	
binding site 3 antisense:	5'-CCAAGCTAGCCTCACGAG-3'	
binding site 2 sense:	5'-AGAGCGGACAATCAAGA-3'	
binding site 2 antisense:	5'-CATTGATACAGCCGACCA-3'	
binding site 1 sense:	5'-CAAAGGTTCCCTGTTGCCTA-3'	
binding site 1 antisense:	5'-CCAGTTTCAGCGAGGAGCTA-3'	
Primers for *KLF7* promoter construct:		
(-1986/+336) *KLF7* sense:	5'-TATAGAGCTCACATGTAGAAAGGACAAA-3'	SacI
(-1257/+336) *KLF7* sense:	5'-TATAGAGCTCGACTACAGGCGTGAGCCA-3'	SacI

(-1055/+336) *KLF7* sense:	5’-TATAGAGCTCAAAACCTGCTTCTGACAT-3’	SacI
(-130/+336) *KLF7* sense:	5’-TATAGAGCTCTACTGACACTTTAGGTGC-3’	SacI
Antisense:	5’-ATATCTCGAGGTCTCGGTGAATTAACCA-3’	XhoI
Primers for *KLF7* promoter site-directed mutagenesis:		
*B*inding site d (NF-κB binding site)		
Binding site d mutation sense:	5’-TTGCAGTACAGAG**gagc**TCCATCAAATCAA-3’	
Binding site d mutation antisense:	5’-TTGATTTGATGGA**gctc**CTCTGTACTGCAA-3’	
*B*inding site c (STAT3 binding site)		
Binding site c mutation sense:	5’-TACTGGCTTCTTT**gatc**AAATCTTTGTCCAG-3’	
Binding site c mutation antisense:	5’-CTGGACAAAGATTT**gatc**AAAGAAGCCAGTA-3’	
*B*inding site b (NF-κB binding site)		
Binding site b mutation sense:	5’-TAGTGCTTGCTGG**tgca**TCCCTACCATTGG-3’	
Binding site b mutation antisense:	5’-CCAATGGTAGGGA**tgca**CCAGCAAGCACTA-3’	
*B*inding site a (AP-1 binding site)		
Binding site a mutation sense:	5’-ATATGTTGAATG**ctga**ATTGAATTCAA-3’	
Binding site a mutation antisense:	5’-TTGAATTCAAT**tcag**CATTCAACATAT-3’	
Primers used for ChIP in the *KLF7* promoter:		
distant region sense:	5’-GCCTGTTATTATATCACC-3’	
distant region antisense:	5’-TCAGAATTTAAGGCACT-3’	
binding site sense:	5’-CTGGCTTCACAACTATCTGG-3’	
binding site antisense:	5’-CCCCATCTAAGAATAGCAT-3’	

Table S9. Knockdown shRNA sequences used in this study

Gene name	Sequence
KLF7 shRNA-1	CTTGAATTGGAACGCTACCTA
KLF7 shRNA-2	GCTACTTCTCAGCTTTACCAT
KLF7 shRNA-3	CCACATGAAGAGACATATCTA
TLR4 shRNA-1	GCCACCTCTCTACCTTAATAT
TLR4 shRNA-2	CCCTGCTGGATGGTAAATCAT
TLR4 shRNA-3	GTGGTTCCTAATATTACTTAT
PTK2 shRNA-1	CGTGAAGATGTGGTCCTGAAT
PTK2 shRNA-2	GAGAGCATGAAGCAAAGAATT
PTK2 shRNA-3	CCGGTCGAATGATAAGGTGTA
RAGE shRNA-1	CGAGTCCGTGTCTACCAGATT
RAGE shRNA-2	GCGGCTGGAATGGAAACTGAA
RAGE shRNA-3	CCGTGCTGTCAGCATCAGCAT
p65 shRNA-1	CGGATTGAGGAGAAACGTAAA
p65 shRNA-2	CACCATCAACTATGATGAGTT
p65 shRNA-3	CCTGAGGCTATAACTCGCCTA
KLF5 shRNA	CCTATAATTCCAGAGCATAAA
KLF8 shRNA	CCCAGCACTGTTTAATGACAT
KLF13 shRNA	CGGGCGAGAAGAAGTTCAGCT

Reference:

1. Huang W, Chen Z, Shang X, Tian D, Wang D, Wu K, et al. Sox12, a direct target of FoxQ1, promotes hepatocellular carcinoma metastasis through up-regulating Twist1 and FGFBP1. Hepatology. 2015; 61: 1920-33.
2. Li W, Li Y, Siraj S, Jin H, Fan Y, Yang X, et al. FUN14 Domain-Containing 1-Mediated Mitophagy Suppresses Hepatocarcinogenesis by Inhibition of Inflammasome Activation in Mice. Hepatology. 2019; 69: 604-21.
3. Tang Z, Kang B, Li C, Chen T, Zhang Z. GEPIA2: an enhanced web server for large-scale expression profiling and interactive analysis. Nucleic Acids Res. 2019; 47: W556-W60.
4. Nagy A, Munkacsy G, Gyorffy B. Pancancer survival analysis of cancer hallmark genes. Sci Rep. 2021; 11: 6047.
